# Joint X-ray/NMR structure refinement of multidomain/multisubunit systems

**DOI:** 10.1007/s10858-018-0212-3

**Published:** 2018-10-11

**Authors:** Azzurra Carlon, Enrico Ravera, Giacomo Parigi, Garib N. Murshudov, Claudio Luchinat

**Affiliations:** 1 Magnetic Resonance Center (CERM) and Interuniversity Consortium for Magnetic Resonance of Metallo Proteins (CIRMMP), Via L. Sacconi 6, 50019 Sesto Fiorentino, Italy; 2 Department of Chemistry “Ugo Schiff”, University of Florence, Via della Lastruccia 3, 50019 Sesto Fiorentino, Italy; 3 MRC Laboratory for Molecular Biology, Francis Crick Ave, CB2 0QH Cambridge, UK

**Keywords:** Structure refinement, Residual dipolar couplings, Integrated structural biology, REFMAC

## Abstract

Data integration in structural biology has become a paradigm for the characterization of biomolecular systems, and it is now accepted that combining different techniques can fill the gaps in each other’s blind spots. In this frame, one of the combinations, which we have implemented in REFMAC-NMR, is residual dipolar couplings from NMR together with experimental data from X-ray diffraction. The first are exquisitely sensitive to the local details but does not give any information about overall shape, whereas the latter encodes more the information about the overall shape but at the same time tends to miss the local details even at the highest resolutions. Once crystals are obtained, it is often rather easy to obtain a complete X-ray dataset, however it is time-consuming to obtain an exhaustive NMR dataset. Here, we discuss the effect of including a-priori knowledge on the properties of the system to reduce the number of experimental data needed to obtain a more complete picture. We thus introduce a set of new features of REFMAC-NMR that allow for improved handling of RDC data for multidomain proteins and multisubunit biomolecular complexes, and encompasses the use of pseudo-contact shifts as an additional source of NMR-based information. The new feature may either help in improving the refinement, or assist in spotting differences between the crystal and the solution data. We show three different examples where NMR and X-ray data can be reconciled to a unique structural model without invoking mobility.

**Electronic supplementary material:**

The online version of this article (10.1007/s10858-018-0212-3) contains supplementary material, which is available to authorized users.

## Introduction

Integrated Structural Biology tries to merge the results from different experimental techniques, making the most of the information encoded in each (Ward et al. 2013; van den Bedem and Fraser 2015; Carlon et al. 2016b; Schlundt et al. 2017). By and large, the two most common experimental techniques for biomolecular structure determination are X-ray diffraction (no. pdb entries retrieved on October 2, 2018: 129581/144682, i.e. 89.6%) and solution NMR spectroscopy (no. pdb entries: 12303/144682, i.e. 8.5%), and single particle cryo electron microscopy (no. pdb entries: 2434/144682, i.e. 1.7%) is progressing rapidly. X-ray crystallographic diffraction (and cryo-EM) data give information that progress from the overall shape of the molecule up to individual atom positions as the resolution increases; on the contrary, NMR data mostly progress from short-range inter-atom distances and bond orientations to the overall shape of the molecule with increasing number and quality of restraints. The combination of these techniques thus yields valuable information throughout the whole range of distances, even in the presence of suboptimal X-ray and/or NMR data.

It is often the case that the X-ray structures and the NMR data are not in perfect agreement with each other. Quite often, these inconsistencies are interpreted as significant and subsequently reconciled by considering structural rearrangements on passing from solid state to solution and/or invoking mobility of different extents and on different timescales (Chou et al. 2001; Bertini et al. 2004, 2009; Volkov et al. 2006; Tang et al. 2007; Lange et al. 2008; Cerofolini et al. 2013; Fenwick et al. 2014; Carlon et al. 2016a, b; Andrałojć et al. 2017). However, before following this path, one should consider first if the X-ray data has sufficient information to position atoms and bonds that are observed by NMR, because, if placed with a low accuracy, inconsistencies may lose significance (structural noise) (Zweckstetter and Bax 2002).

X-ray data, NMR-derived distance restraints and backbone dihedral angles were previously used for a joint refinement of protein structure (Shaanan et al. 1992). It was noticed that crystal models frequently present a large number of NOE distance violations, as well as solution models obtained by NMR poorly fit the X-ray data: these discrepancies may either be due to differences in the molecular structure between solution and solid state, or to the different but complementary information contained in these two types of data. The fact that X-ray and NMR data can be combined to produce models that are compatible with both sets of data, in the sense that (i) the crystallographic R-factor and the free R-factor are the same to those calculated with X-ray data alone, and that (ii) the NMR restraint violations are minimal, indicates that the discrepancies between solution and crystal structures may be apparent rather than real, and that a joint refinement against all data may provide a more reliable model than those obtained using one of the techniques alone. On the other hand, the violating restraints in the joint refinement may provide an indication of the regions where real differences occur.

Joint refinements against X-ray and NMR data were previously performed using distance restraints and backbone dihedral angles as NMR restraints, through the programs CNS and X-PLOR (Brunger et al. 1987). The calculations indicated that the two sets of data are consistent for a number of studied proteins (interleukin-1 β (Shaanan et al. 1992), bovine pancreatic trypsin inhibitor (Schiffer et al. 1994), p53 (Miller et al. 1996), HU (Raves et al. 2001), the integral membrane protein complex DsbB-DsbA (Tang et al. 2011), mostly improving the geometry of the model in terms of Ramachandran plot with respect to the structure calculated without NMR data. In some cases, the joint refinement clearly provided more accurate models, for instance in the presence of regions poorly determined by X-ray data alone, due to packing disorder within the crystal [L9 protein (Hoffman et al. 1996)], or with low- to medium-resolution diffraction data [maltose binding protein-L30e fusion protein in complex with RNA (Chao and Williamson 2004)].

Paramagnetic data such as pseudocontact shifts (PCSs) and residual dipolar couplings (RDCs) carry long-range information on the relative position—or internuclear vector orientation—of the NMR-active nuclei in a protein with respect to a common frame, and thus result very useful to validate and refine the global fold of a protein in solution when crystallographic data are available.

RDCs that originate from partial alignment due to either interaction with an external alignment medium (Tolman et al. 1995) or from the presence of a paramagnetic centre (Banci et al. 1998b), are extremely sensitive reporters of the angles between internuclear vectors and the principal axis frame of the alignment tensor. When RDCs are obtained because of the presence of a paramagnetic centre (Bertini et al. 2002a; Volkov et al. 2006; Clore 2011; Koehler and Meiler 2011; Knight et al. 2013; Hass and Ubbink 2014; Nitsche and Otting 2017; Ravera et al. 2017), PCSs also arise and provide information about the positions of nuclei in the Cartesian space defined by the principal axis frame of the magnetic susceptibility tensor associated to the paramagnetic centre (Kurland and McGarvey 1970; Banci et al. 1996; Bertini et al. 2002a, b). Since the alignment is caused by the anisotropy of the magnetic susceptibility, the two experimental datasets are characterized by the same tensor, and PCSs can be used to strengthen the estimate of the tensor to be used for fitting the RDCs (Bertini et al. 2004, 2009).

PCSs and RDCs contain structural information that has proved very helpful for solving protein structures (Gochin and Roder 1995; Banci et al. 1996, 1998b; Bertini et al. 2001; Gaponenko et al. 2004; Diaz-Moreno et al. 2005; Jensen et al. 2006; Schmitz et al. 2012), and they have therefore been included as structural restraints in the most commonly used programs for protein structure determination from NMR data (Banci et al. 1998a, b, 2004; Schwieters et al. 2003; Schmitz and Bonvin 2011; Schmitz et al. 2012).

We have included PCSs and RDCs as structural restraints in the Macromolecular Crystal Structure refinement program—REFMAC5 (Murshudov et al. 1997, 2011) available from CCP4 (Winn et al. 2011). This program uses the maximum-likelihood technique to optimize the fit of atomic model parameters into X-ray crystallographic data. The agreement with X-ray data is monitored through the R-factor and the free R-factor, and agreement with the NMR data is monitored through the Q-factor (Cornilescu et al. 1998). As it is common when using X-ray data for structure refinement, some deviations from the ideal geometry of covalent bonding are allowed, and, for this purpose, appropriate weights of the geometric restraints relative to the NMR and X-ray restraints must be selected. If large deviations from ideal geometry are allowed, full compatibility of crystal and NMR data can be achieved. Our strategy for the joint refinement was based on fixing the weights of the geometric restraints to the values providing free R-factor and geometry parameters close to those obtained without including the NMR data. In summary, the applied protocol consists of two steps:

1. standard refinement with REFMAC5 performed using only X-ray data. The final values obtained for R-factor, free R-factor, chemical bonds violations (RMS BondLength, BondAngle and ChirVolume) are taken as reference values.
2. PCS and/or RDC data are added, together with the geometrical restraints. The weight of PCS, RDC and geometrical restraints are changed until the final values of R-factor, free R-factor, and chemical bonds violations are comparable (or better) than the reference values, and PCSs and/or RDCs are fitted as best as possible. This step is repeated until satisfactory values are reached (Q factor < 0.20), or until results show that X-ray and NMR data are in disagreement.

This approach was previously used to show that single refined structures can be calculated using simultaneously X-ray and NMR data collected for the catalytic domain of the protein matrix metalloproteinase 1, for the protein ubiquitin and for the third IgG-binding domain of protein G, thus indicating that no appreciable structural changes occur when passing from solution to solid state; the backbone RMSD between the structures calculated with and without the NMR data is below 0.03 Å. On the contrary, in the case of the N-terminal domain of calmodulin, the joint refinement does not produce any atomic model in agreement with both X–ray and NMR data, indicating that there are some structural differences between the solution and solid state protein (Rinaldelli et al. 2014).

Biomolecules often comprise several domains and subunits, each potentially experiencing an intrinsic variability, and RDCs and PCSs can provide a precious contribution to the characterization of the interdomain/intersubunit arrangement (Valafar and Prestegard 2004; Pintacuda et al. 2007; Bertini et al. 2009, 2010; Simon et al. 2010; Berlin et al. 2010, 2013; Schmitz and Bonvin 2011; Fragai et al. 2013; Cerofolini et al. 2013; Russo et al. 2013; Rinaldelli et al. 2015). In fact, if the complex is composed of domains/proteins with known crystal structures, which do not change in solution upon complex formation, PCSs and RDCs can be very effective in determining their relative position and orientation, once multiple paramagnetic metal ions have been alternatively coordinated to one protein or attached through a rigid binding tag. The process is rather simple. First the RDCs (and PCSs) of each domain/subunit are calculated from an initial model such as the X-ray structure. If a domain or subunit is rigid all the RDCs (and PCSs) referring to atoms belonging to it should fit to a single tensor. If not, one must conclude that the domain is experiencing some static or dynamic rearrangement (Andrałojć et al. 2015; Carlon et al. 2016a).

The second step requires the comparison of the tensors calculated for each domain. If the spatial arrangement of the domains/subunits is the same between the crystal and the solution, all the tensors from each domain will have the same magnitude. If, on the other hand, the tensors have the same magnitude but different orientations, one can conclude that a static rigid rearrangement has occurred upon crystallization (Carlon et al. 2016a, b). If both magnitude and orientation differ, a dynamic process must be present.

The situation described above may not be fulfilled in the presence of experimental uncertainty and in the case where there is not sufficient data to correctly calculate both the tensor and the atomic coordinates. Therefore, the best-fit values of back-calculated tensors may appear more different than needed to be consistent with the experimental data. In this case, one can decide to impose a constraint in the refinement and check whether the agreement between the experimental and calculated NMR data improves or becomes worse. In the first case, the constraint will have helped in improving the refinement, in the latter case it will have assisted in spotting differences between the crystal and the solution data.

We have thus modified the REFMAC-NMR program for either constraining the alignment tensors to a unique orientation or to a unique set of principal values. In molecular structural refinements against both X-ray and RDC data (possibly complemented by PCS data), there are several cases where constraining reciprocal orientation and/or magnitude of tensors may be useful. Examples that can be easily encountered can be grouped into two cases:

- rigid systems composed of multiple subunits, where RDCs have been measured in different samples to reduce spectral complexity: in this case the reciprocal orientations of the tensors must be fixed, whereas their magnitude can vary because of slight differences in the concentration of the alignment medium;
- rigid systems composed of multiple domains, where the relative orientations of the domains in solution are expected to be different from the solid state: in these cases the reciprocal orientations of the tensors must be allowed to vary, whereas their magnitude is fixed.

We have applied the new program for the refinement of three systems composed of multiple units belonging to these cases. In all three cases we have found that it is possible to fit the data to a single structural model, without invoking mobility to explain the inconsistency between the NMR data and the X-ray structures.

## Methods

All refinement calculations were performed using REFMAC 5.9.0000 (Murshudov et al. 2011; Kovalevskiy et al. 2018), in which an option to constrain the relationship between different tensors have been introduced (see results and discussion for the details about implementation). The refinement protocol applied for the simultaneous refinement using NMR and X-ray is detailed in Rinaldelli et al. (2014).

In the most general form, the effect of residual dipolar coupling (RDC) is described as follows (Bertini et al. 2002b):

$$RDC=3k\left[ {{S_{zz}}\frac{{2z_{{AB}}^{2} - x_{{AB}}^{2} - y_{{AB}}^{2}}}{{2r_{{AB}}^{2}}}+\left( {{S_{xx}} - {S_{yy}}} \right)\frac{{x_{{AB}}^{2} - y_{{AB}}^{2}}}{{2r_{{AB}}^{2}}}+{S_{xy}}\frac{{2{x_{AB}}{y_{AB}}}}{{r_{{AB}}^{2}}}+{S_{xz}}\frac{{2{x_{AB}}{z_{AB}}}}{{r_{{AB}}^{2}}}+{S_{yz}}\frac{{2{y_{AB}}{z_{AB}}}}{{r_{{AB}}^{2}}}} \right]$$

with

$$k= - \frac{{{\mu _0}{S_{LS}}}}{{4\pi }}\frac{{{\gamma _A}{\gamma _B}\hbar }}{{2\pi r_{{AB}}^{3}}}$$

$$x_{{AB}}^{2}={\left( {{x_A} - {x_B}} \right)^2},\quad y_{{AB}}^{2}={\left( {{y_A} - {y_B}} \right)^2},\quad z_{{AB}}^{2}={\left( {{z_A} - {z_B}} \right)^2},\quad r_{{AB}}^{2}=x_{{AB}}^{2}+y_{{AB}}^{2}+z_{{AB}}^{2}$$

where *r*_*AB*_ is the distance between the two coupled nuclei A and B, and *s*_*ij*_ are the components of the molecular alignment tensor. The anisotropies of the alignment tensor are described by the fraction of alignment along the z-axis (*A*) and by the rhombicity (*η*) as follows:

$$A=\frac{3}{2}{\tilde {S}_{zz}}$$

$$\eta =\frac{{{{\tilde {S}}_{xx}} - {{\tilde {S}}_{yy}}}}{A}$$

when ${\tilde {S}_{ii}}$ are the components of the alignment tensor in the frame in which it is diagonal.

In the case that the alignment of the molecule is induced by magnetic susceptibility anisotropy, the alignment is dictated by the anisotropy of the magnetic susceptibility tensor $\varvec{\chi}$ and the tensor components are expressed as follows:

$${\tilde {S}_{ii}}=\frac{{B_{0}^{2}}}{{15{\mu _0}kT}}\left( {{{\tilde {\chi }}_{ii}} - \bar {\chi }} \right)$$

where ${\tilde {S}_{ii}}$ are the principal components of the alignment tensor ***S***, ${\tilde {\chi }_{ii}}$ are the principal components of the magnetic susceptibility tensor $\varvec{\chi}$, and $\bar {\varvec{\chi}}$ is one-third of the trace of the tensor $\varvec{\chi}$ (note that only the anisotropic components of the tensor should be considered), and it is common to define $\Delta {\chi _{ax}}=\frac{3}{2}{\tilde {\chi }_{zz}}$ and $\Delta {\chi _{rh}}=\frac{{{{\tilde {\chi }}_{xx}} - {{\tilde {\chi }}_{yy}}}}{2}$.

If the magnetic susceptibility anisotropy is induced by paramagnetic metals, PCSs are usually also measured. PCSs are related to the nuclear position according to the following equation (Bertini et al. 2002b):

$$PCS=\frac{1}{{4\pi r_{{AM}}^{3}}}\left[ {\left( {{\chi _{zz}} - \bar {\chi }} \right)\frac{{2z_{{AM}}^{2} - x_{{AM}}^{2} - y_{{AM}}^{2}}}{{2r_{{AM}}^{2}}}+\left( {{\chi _{xx}} - {\chi _{yy}}} \right)\frac{{x_{{AM}}^{2} - y_{{AM}}^{2}}}{{2r_{{AM}}^{2}}}+{\chi _{xy}}\frac{{2{x_{AM}}{y_{AM}}}}{{r_{{AM}}^{2}}}+{\chi _{xz}}\frac{{2{x_{AM}}{z_{AM}}}}{{r_{{AM}}^{2}}}+{\chi _{yz}}\frac{{2{y_{AM}}{z_{AM}}}}{{r_{{AM}}^{2}}}} \right]$$

Position and orientation of protein domains in solution and in the crystal state were compared as follows: the N-terminal domains of solution and crystal structures are superimposed and the distance between the C-terminal domains in the two states is evaluated in terms of the distance between their centres of mass, and of the angular deviation between their orientations. The latter is calculated as the angle *θ* obtained from the rotation matrix *R* which brings the C-terminal domain of one structure to be superimposed to the C-terminal domain of the other structure, according to the following equation:

$$\theta ={\text{arcos}}\frac{{{\text{tr}}(R) - 1}}{2}$$

The back-calculation of tensor parameters and the rigid body minimizations were performed using the FANTEN web application (Rinaldelli et al. 2015). Comparison of the tensors back-calculated independently for different structural units is done according to two criteria (three for paramagnetic cases): tensor magnitude, tensor alignment, and metal distance for paramagnetic cases (Russo et al. 2013). Comparison of the tensor magnitude is obtained from the ratio of their axial components of as following:

$$tensor\;magnitude = \frac{{\tilde {S}_{{zz}}^{1}}}{{\tilde {S}_{{zz}}^{2}}}$$

where values close to 1 indicate very similar tensor magnitude. Comparison of the tensor alignment (and shape) is performed by calculating the normalized dot product of the five independent components constituting the Saupe matrices (*S*) of the two tensors as following:

$$tensor\;alignment= \frac{{\overline {{{S^1}}} \cdot \overline {{{{\tilde {S}}^2}}} }}{{\left\| {{S^1}} \right\| \cdot \left\| {{S^2}} \right\|}}$$

where values close to 1 indicate very similar tensor alignment. Metal distances are calculated as geometric distances between the average position of paramagnetic metals, as obtained after the refinement calculation, and the position of each lanthanoid as back-calculated from the C-terminal domain.

Experimental reflections are taken from the corresponding PDB entry, whereas the NMR data are available in the original publications.

## Results and discussion

### Program implementation

In the previous version of REFMAC-NMR (Rinaldelli et al. 2014), the alignment tensor ***S*** describing the RDCs (or the $\varvec{\chi}$ tensor describing the PCSs) was determined through a Gauss–Newton optimization approach of 5 tensor components *S*_*zz*_, *S*_*xx*_, *S*_*yy*_, *S*_*xy*_, *S*_*xz*_, *S*_*yz*_. Using these components as fitting parameters, application of constraints is rather involved. Therefore, we have re-parameterized the problem to allow for the inclusion of such constraints.

Instead of using the 5 tensor components given above, the tensor ***S*** is reconstructed as follows: the orientation is provided by three variables describing the rotation that brings the tensor ***S*** from any arbitrary molecular frame to the frame where it is diagonal, and the magnitude is determined by two of the diagonal elements, $${\tilde {S}_{xx}}$$ and $${\tilde {S}_{yy}}$$$$\left( {{{\tilde {S}}_{zz}}= - \,{{\tilde {S}}_{xx}} - {{\tilde {S}}_{yy}},\,{\text{as the tensor is defined as traceless}}} \right)$$. For a convenient sampling of the orientational space and to simplify the handling of the derivatives, the rotation is expressed in terms of quaternions:

$${\varvec{q}}= {q_0}+{q_1}+{q_2}+{q_3}= \cos \frac{\theta }{2}+({v_1}{\varvec{i}}+{v_2}{\varvec{j}}+{v_3}{\varvec{z}})\sin \frac{\theta }{2}$$

where ***i, j, k*** are unit vectors representing the three Cartesian axes, $v_1, v_2, v_3$ are the components of the unit vector defining the axis of rotation, and $\theta$ is the angle of rotation. To represent a rotation, quaternions must satisfy the constraint $\left| {\varvec{q}} \right|=1$ (unit quaternions). This reduces the number of independent variables describing the rotation matrix to three. Thus, the new parameters used in the calculations are the following:

$$p=\left[ {{q_0}, {q_1}, {q_2}, {q_3},\,{{\tilde {S}}_{xx}}, {{\tilde {S}}_{yy}}} \right] \;with\;\left| {\varvec{q}} \right|=1.$$

The minimization performed by REFMAC for the structure refinement requires the computation of the first and second derivatives with respect to the selected parameters of the target function *f* to be minimized, reported in “Problem re-parameterization“ in Appendix.

In the presence of two structural units, two independent tensors can be defined, ***S***_*a*_ and ***S***_*b*_, depending on the two sets of the parameters *p*_*a*_ and *p*_*b*_ defined as:

$${p_a}=\left[ {{q_{a,i}},{{\tilde {S}}_{a,ii}}} \right],\quad {p_b}=\left[ {{q_{b,i}},{{\tilde {S}}_{b,ii}}} \right] \;\text{with}\;i=0, 1, 2, 3\;\text{and}\;ii=xx, yy$$

The function *f* is thus the sum of the functions referring to each domain:

$$f\left( {{p^\prime }} \right)=f\left( {{p_a},{p_b}} \right)={f_a}\left( {{p_a}} \right)+{f_b}\left( {{p_b}} \right)={f_a}\left( {{q_{a,i}},{{\tilde {S}}_{a,ii}}} \right)+{f_b}\left( {{q_{b,i}},{{\tilde {S}}_{b,ii}}} \right)$$

where

$${p^\prime }=\left[ {{p_a},{p_b}} \right]=\left[ {{q_{a,i}},{{\tilde {S}}_{a,ii}},{q_{b,i}},{{\tilde {S}}_{b,ii}}} \right]$$

The first and second derivatives of *f* are given by the derivatives of *f*_*a*_ with respect to the *p*_*a*_ parameters and of *f*_*b*_ with respect to the *p*_*b*_ parameters, and are reported in the “Imposing anisotropy and orientation constraints between tensors” in Appendix.

In order to constrain the *orientation* of the two tensors to be same (${q_{a,i}}={q_{b,i}}$ for *i* = 0, 1, 2, 3), parameters and target function can be re-expressed in the following way:

$${p^\prime }=\left[ {{q_i},{{\tilde {S}}_{a,ii}},{{\tilde {S}}_{b,ii}}} \right]\quad f({p^\prime })={f_a}\left( {{q_i},{{\tilde {S}}_{a,ii}}} \right)+{f_b}\left( {{q_i},{{\tilde {S}}_{b,ii}}} \right)$$

where the non-null elements of the first and second derivatives of *f* for the present case are reported in “Imposing anisotropy and orientation constraints between tensors” in Appendix.

Likewise, in order to constrain the *anisotropy values* of the two tensors to be the same (${\tilde {S}_{a,ii}}={\tilde {S}_{b,ii}}$ for *ii* = *xx, yy*), parameters and target function can be re-expressed in the following way:

$${p^\prime }=\left[ {{q_{a,i}},{{\tilde {S}}_{ii}},{q_{b,i}}} \right] \quad f\left( {{p^\prime }} \right)={f_a}\left( {{q_{a,i}},{{\tilde {S}}_{ii}}} \right)+{f_b}\left( {{q_{b,i}},{{\tilde {S}}_{ii}}} \right)$$

where the non-null elements of the first and second derivatives of *f* are given in “Imposing anisotropy and orientation constraints between tensors” in Appendix.

Analogous considerations can be done for the tensor $\varvec{\chi}$, used when PCSs are used.

The constraints described above have been implemented in REFMAC-NMR and can be applied to any generic set of tensors, according to the standard instruction file provided to REFMAC-NMR (see some examples in the Supplementary Materials). The algorithm can thus identify the tensors to be constrained (in orientation, anisotropy values, or both) according to user’s instructions, and perform the appropriate minimizations.

### Using tensor constraints in REFMAC-NMR refinement

#### Tensor orientation in rigid multisubunit systems

To reduce the spectral complexity, the different subunits of a multisubunit systems may be separately expressed and reconstituted in vitro, labelling them differentially. This approach might introduce slight perturbations when recording RDCs with external alignment media: since the RDCs are collected in different samples, the concentration of the alignment medium may vary from one sample to the other, and this might simulate of protein mobility. We have recently encountered such a situation in the refinement of the complex between Sxl and CSD1 against X-ray and diamagnetic RDC data (Hennig et al. 2014; Carlon et al. 2016b). The refinement calculations were performed using a single alignment tensor for the complete RDC data and scaling the CSD1 RDC values by an empirical factor 0.8. This scaling factor was obtained from the comparison of the axial anisotropies obtained from the refinements performed for two proteins using two independent tensors.

Here we re-examine this system by imposing the tensors from the individual subunits to be equally oriented. A slightly different concentration of the alignment medium, in fact, affects the magnitude of the alignment tensors, but not their orientation. The inclusion of this orientation constraint represents the safest approach for an optimal refinement and permits the identification of the scaling factor between the different experimental conditions. We have thus refined again the structure of the complex between Sxl and CSD1 against the X-ray and diamagnetic RDC data using two alignment tensors constrained to have the same orientation.

The result of the refinements of the individual Sxl and CSD1 domains performed with either a single tensor (and uniform scaling of the CSD1 RDC values by an empirical factor 0.8), as previously done, or with the inclusion of the orientation constraint between the tensors of the two domains, now allowed by REFMAC-NMR, are compared in terms of REFMAC-NMR output and tensor comparisons (Table 1 and S1). A slight increase in the quality of the refinement, in terms of smaller R-free and Q-factor, is present when the orientation constraint is applied.

**Table 1:** REFMAC-NMR output for the independent refinement of Sxl and CSD1 domains, before and after the inclusion of the orientation constraint for the tensors calculated for the individual units

PDB code: 4QQB; resolution: 2.80 Å				
Parameters	Original structure	No constraint^a^		Constraint
		−NMR	+NMR^b^	+NMR
R-value	0.198	0.1968	0.1986	0.1988
**R-free**	**0.236**	**0.2352**	**0.2362**	**0.2360**
RMSD bond length	0.006	0.0064	0.0101	0.0100
RMSD bond angles	1.113	1.2566	1.6739	1.6729
RMSD chiral volume	0.074	0.0967	0.1067	0.1070
**Q-factor RDC**	**0.440**	–	**0.131**	**0.121**

R-free and the Q-factor of the RDC fit are in bold as the most informative parameters

Refinement are performed with REFMAC-NMR 5.9.000 version

^a^Values are different from those in (Carlon et al. 2016b), calculated with REFMAC-NMR 5.8.0073 version instead of REFMAC-NMR 5.9.000 version

^b^Uniform scaling of the CSD1 RDC values by an empirical factor 0.8

#### Tensor magnitude in rigid multidomain systems

There are cases in which a multidomain protein experiences different reciprocal arrangements of its domains upon binding to targets of different size. This is for instance the case of calmodulin (CaM) (Kursula 2014), the prototypical calcium sensor, upon binding to the death-associated protein kinase (DAPk, pdb code: 2X0G) (de Diego et al. 2010) or to a peptide that is derived from it (pdb code: 1YR5) (Bertini et al. 2009). DAPk is much bigger than CaM, whereas the fragment peptide is smaller. When crystallized in the peptide-bound form, the two domains of CaM experience crystal packing forces, which are mitigated when the crystallization occurs in the presence of the whole DAPk. For this system, paramagnetic NMR data (PCSs and RDCs) collected for CaM in the peptide-bound state were found in disagreement with the crystal structure. A solution structure was thus calculated, showing that the peptide-bound CaM adopts a more extended conformation in solution with respect to the crystal (Bertini et al. 2009). Differently, in the case of CaM bound to IQ peptide (pdb code: 2BE6), the agreement between the X-ray structure and paramagnetic NMR data is already quite remarkable (Russo et al. 2013). However, since three different conformations are observed in the crystals, in (Russo et al. 2013) the NMR data were analysed invoking interdomain mobility.

We here analyse the cases of CaM when bound to the DAPk peptide and to the IQ peptide again, through a join refinement including X-ray data and the available paramagnetic NMR data, and making use of the constraint that forces the tensors of the two domains to have the same magnitude and rhombicity, but allowing for different orientations. In this way, even if the domain arrangement in solution is different from the solid state, the PCS and RDC data can be reconciled with the X-ray data, because the different orientation of the tensors reports on the different relative orientation of the protein domains in solution. Following the REFMAC-NMR refinement, a rigid body minimization was performed [using FANTEN web application (Rinaldelli et al. 2015)] in order to recover the relative arrangement of the protein domains in solution (i.e., by matching orientation and origin of the tensors of the two domains). For the calculation of both the DAPk-peptide-bound CaM and DAPk-bond form the NMR data were the same, referring to the peptide-bound sample.

**Fig. 1:**
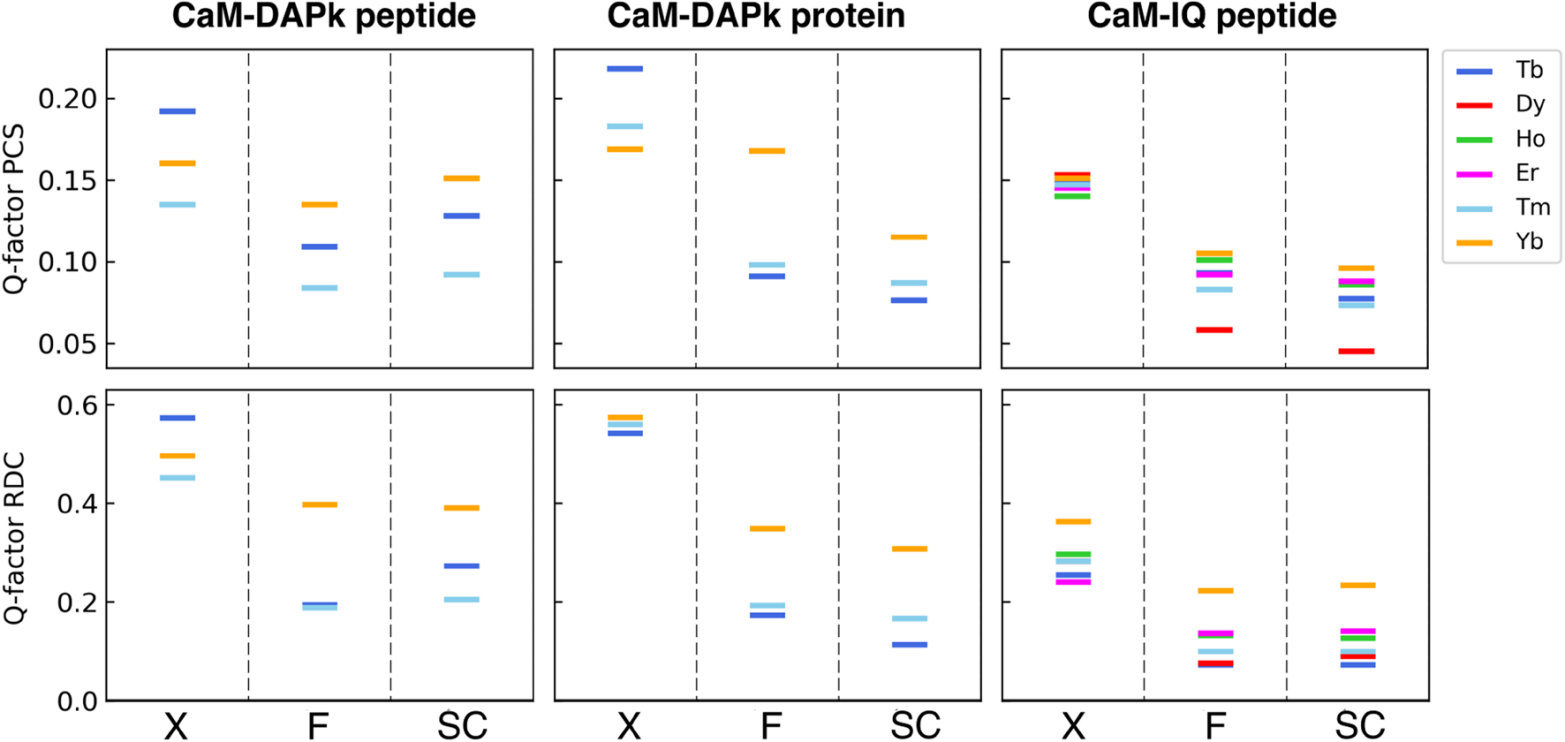
Agreement between observed and calculated data for the considered structures of CaM. Q-factors of PCSs and RDCs for the N- and C-terminal domains are calculated for the original X-ray structures (X), for the structures refined by REFMAC-NMR (F), and for the structures refined using the tensor magnitude constraint after applying rigid body minimization (SC)

**Fig. 2:**
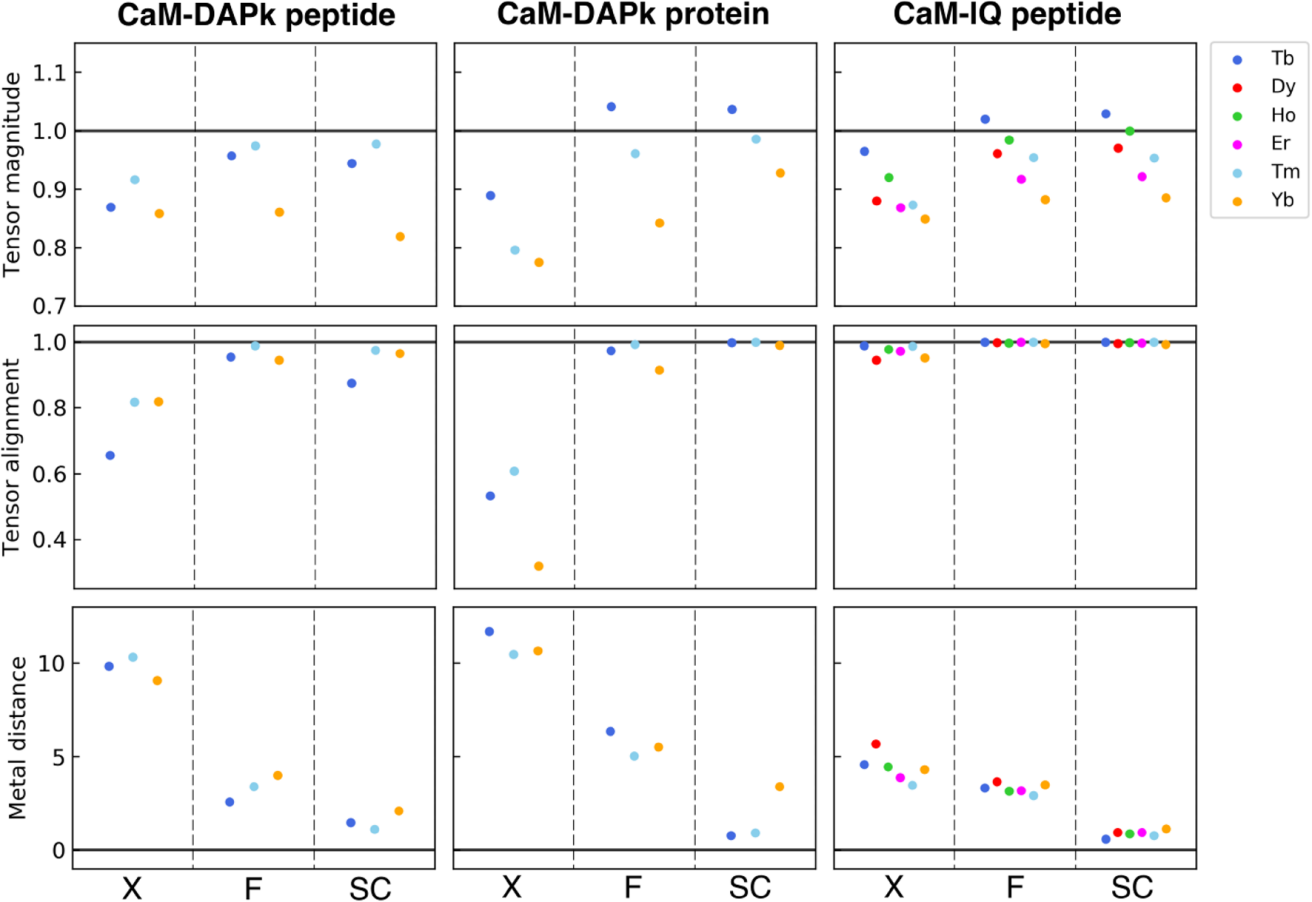
Comparison between the tensor calculated independently for the N- and C-terminal domains in terms of tensor sizes, tensor alignments, and metal positions for the original X-ray structures (X), for the structures refined by REFMAC-NMR (F), and for the structures refined using the tensor magnitude constraint after applying rigid body minimization (SC)

The quality of the refinements was evaluated in terms of (a) overall agreement with the experimental data (R-value, R-free, Q-factor PCS, Q-factor RDC) and with the ideal geometries (Tables 2, 3, 4) (Fig. 1); (b) agreement between experimental and back-calculated NMR data, in terms of residue-specific variations (Figs. S1–S3), and (c) agreement between the tensor parameters back-calculated for each domain (Fig. 2). The latter parameters, obtained with a best fit of the PCS and RDC data against each protein domain independently, reflect the tendency for the system to agree with a single rigid structure. For a single rigid structure, in fact, the magnitude and orientation of the tensors of the different domains should match each other and the positions of the paramagnetic metals should correspond. These quality factors were evaluated for the original X-ray structures (“X” in Figs. 1, 2), for the structures refined with a single tensor for each metal (“F” in Figs. 1, 2), and for the structures refined using the tensor magnitude constraint followed by rigid body minimization of domain positions (“SC” in Figs. 1, 2).

**Table 2:** REFMAC-NMR output for the refinement of 1YR5, before and after the inclusion of the magnitude constraint for the tensors calculated for the individual domains

PDB code: 1YR5; resolution: 1.7 Å				
Parameters	Original X-ray structure		Full-length protein refinement	Individual domain refinement using constrain
		−NMR	+NMR	+NMR
R-value	0.2689	0.2237	0.2270	0.2254
**R-free**	**0.2993**	**0.2815**	**0.2836**	**0.2827**
RMSD bond length	0.0191	0.0123	0.0187	0.0193
RMSD bond angles	1.4455	1.5379	2.1931	2.0110
RMSD chiral volume	0.0925	0.0928	0.1519	0.1571
**Q-factor PCS**	**0.157**	**0.162**	**0.111**	**0.123**
**Q-factor RDC**	**0.533**	**0.533**	**0.212**	**0.203**
Weight matrix	–	0.005	0.005	0.005
Pep2	–	1.0	1.0	1.0

R-free and the Q-factor of the NMR fit are in bold as the most informative parameters

Refinement are performed with REFMAC-NMR 5.9.000 version

**Table 3:** REFMAC-NMR output for the refinement of 2X0G, before and after the inclusion of the magnitude constraint for the tensors calculated for the individual domains

PDB code: 2X0G; resolution: 2.20 Å				
Parameters	Original X-ray structure		Full-length protein refinement	Individual domain refinement using constrain
		−NMR	+NMR	+NMR
R-value	0.2858	0.2224	0.2259	0.2256
**R-free**	**0.3385**	**0.2920**	**0.2934**	**0.2928**
RMSD bond length	0.0206	0.0146	0.0161	0.0138
RMSD bond angles	1.6453	1.8049	1.8869	1.8258
RMSD chiral volume	0.0992	0.1483	0.1563	0.1417
**Q-factor PCS**	**0.179**	**0.190**	**0.126**	**0.098**
**Q-factor RDC**	**0.524**	**0.549**	**0.196**	**0.149**
Weight matrix	–	0.0012	0.0012	0.0012
Pep2	–	0.8	0.8	0.8

R-free and the Q-factor of the NMR fit are in bold as the most informative parameters

Refinement are performed with REFMAC-NMR 5.9.000 version

**Table 4:** REFMAC-NMR output for the refinement of 2BE6, model A, before and after the inclusion of the magnitude constraint for the tensors calculated for the individual domains

PDB code: 2BE6; resolution: Å				
Model A				
Parameters	Original X-ray structure		Full-length protein refinement	Individual domain refinement using constrain
		−NMR	+NMR	+NMR
R-value	0.2802	0.2205	0.2191	0.2192
**R-free**	**0.2773**	**0.2490**	**0.2518**	**0.2508**
RMSD bond length	0.0337	0.0140	0.0164	0.0170
RMSD bond angles	1.3509	1.8169	1.8886	1.9045
RMSD chiral volume	0.0947	0.1275	0.1415	0.1442
**Q-factor PCS**	**0.162**	**0.151**	**0.078**	**0.067**
**Q-factor RDC**	**0.259**	**0.275**	**0.097**	**0.098**
Weight matrix	–	0.0036	0.0036	0.0036
Pep2	–	0.9	0.9	0.9

R-free and the Q-factor of the NMR fit are in bold as the most informative parameters

Refinement are performed with REFMAC-NMR 5.9.000 version

The refinements for the three structures (1YR5, 2X0G and 2BE6) reveal some crucial different features.

#### Structure refinement of CaM in complex with DAPk

**Fig. 3:**
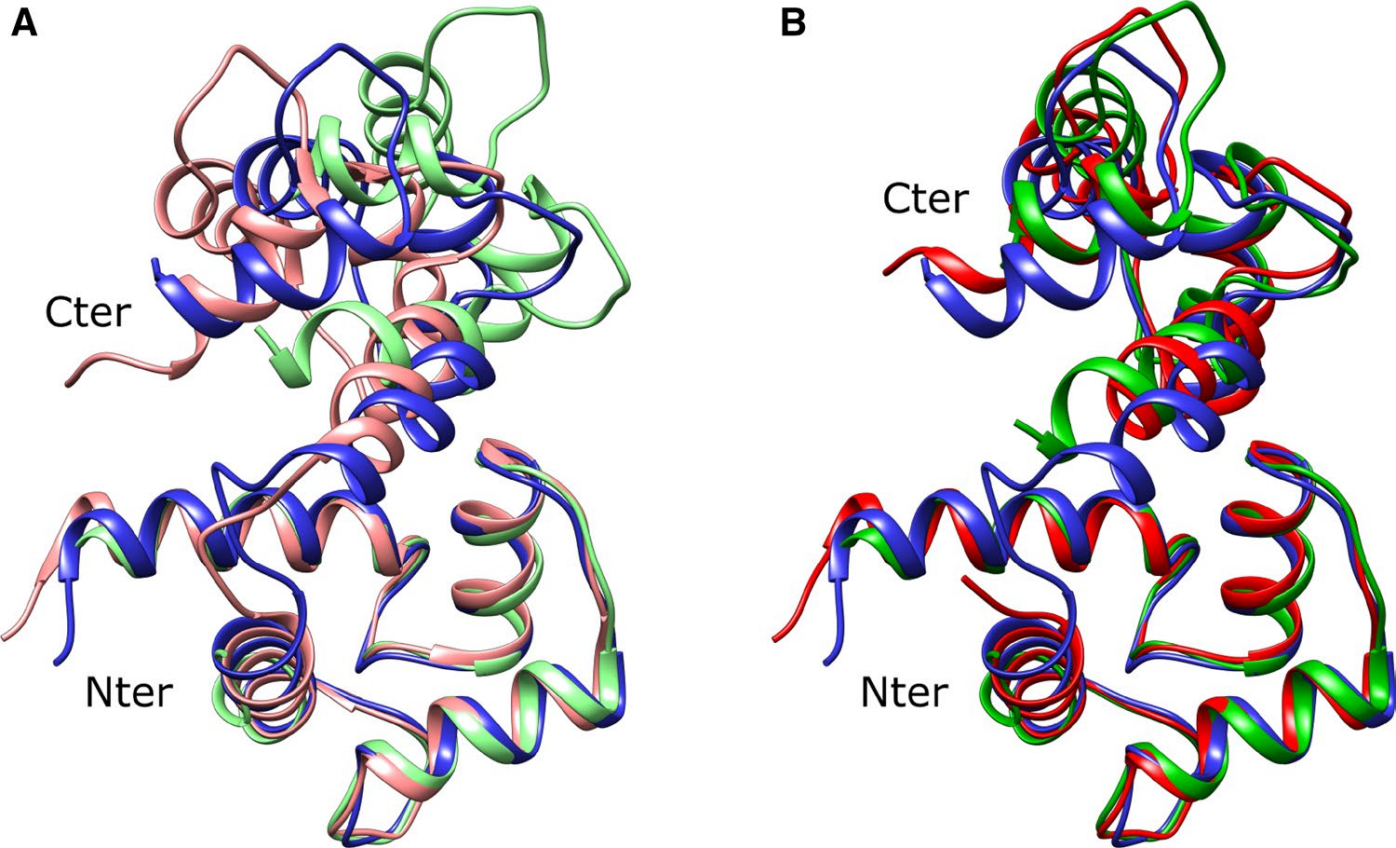
Comparison between the original NMR structure of the peptide-bound CaM (2K61, blue) and **a** the X-ray structures 1YR5 (light red) and 2X0G (light green); **b** the refined 1YR5 (red) and 2X0G (green) structures after rigid body minimization

As previously found, the peptide-bound crystal structure 1YR5 is incompatible with the solution NMR data: even at the point where the agreement of X-ray and ideal geometry is barely acceptable (Table 2), a good fit of the NMR data cannot be obtained (Fig. 1, column “CaM-DAPk peptide”). Furthermore, the back-calculated tensor parameters indicate poor alignment between the tensors calculated for the C-terminal and for the N-terminal domain (Fig. 2, column “CaM-DAPk peptide”). The structure obtained after rigid body minimization becomes closer to the previously found solution structure 2K61 (see Fig. 3): the distance between the centres of mass of the C-terminal domains of the CaM-DAPk peptide structure and of the solution structure decreases from 6.4 to 4.6 Å after the use of rigid body minimization, and the angular deviation (as described in “Materials and methods”) is reduced from 17.1° to 11.5°.

**Fig. 4:**
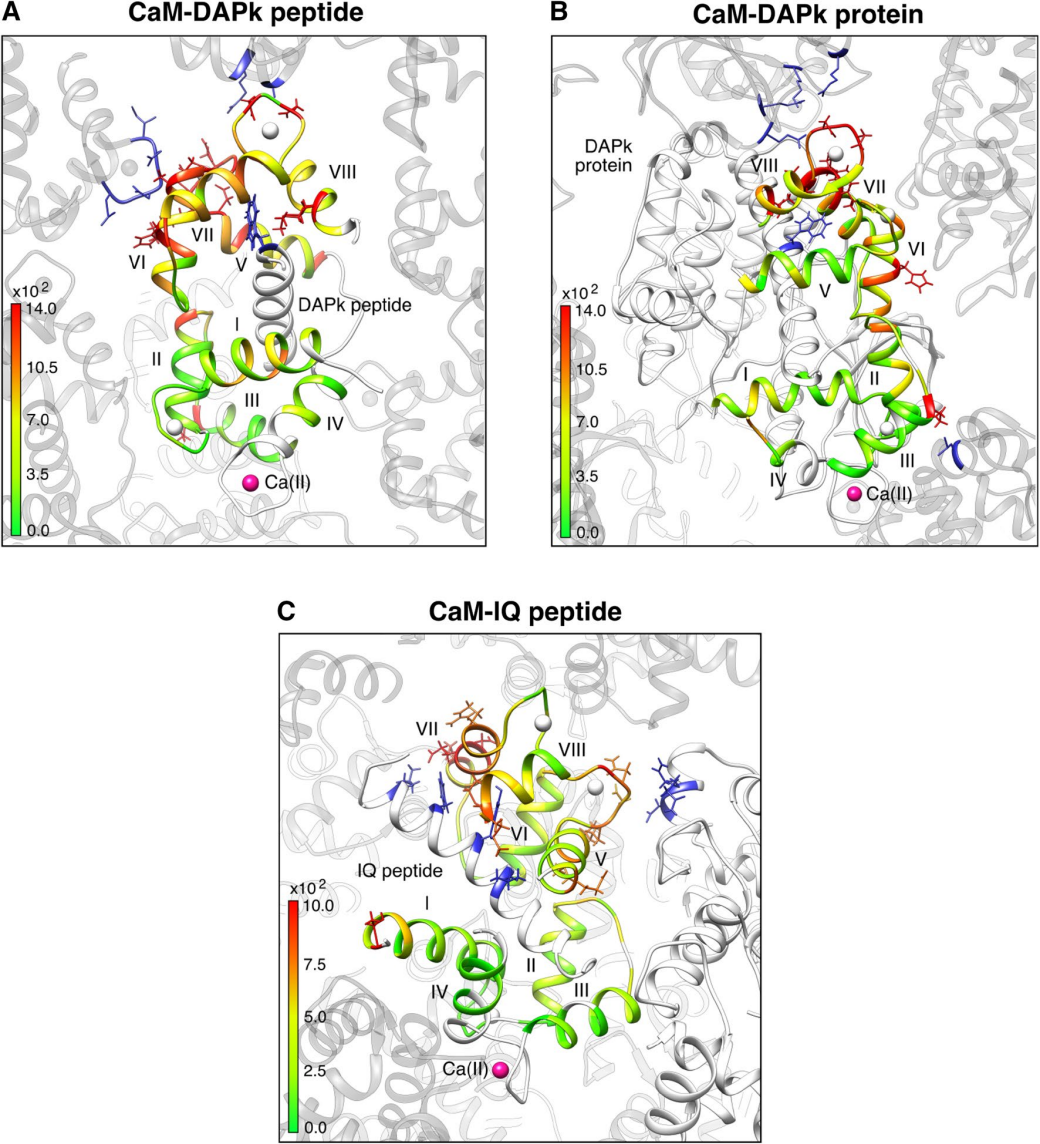
Visualization of intermolecular contacts in the crystal arrangements. PBD codes: **a** 1YR5, **b** 2X0G, **c** 2BE6. Protein residues are coloured according to the differences between experimental and back-calculated PCS values multiplied by *r*^3^ for the refined structures obtained after rigid body minimization

This overall disagreement is easily underpinned by the distribution of the residue-by-residue discrepancy between experimental and back-calculated data (Figs. S1a–S1c). As already reported in a previous work by some of us (Bertini et al. 2009), this is easily explained by the presence of large inter-protein contacts that are present in the crystal (Fig. 4, “CaM-DAPk peptide”). These contacts result to be mostly abolished in the protein-bound crystals (Fig. 4, “CaM-DAPk peptide”) and this is likely due to the larger size of the DAPk protein with respect to CaM and, consistently, the residue-by residue discrepancies are much smaller (Fig. S1a–S1c). As a result, the structure of CaM in complex with the whole DAPk protein results in much better agreement with the solution NMR data collected for the peptide-bound complex, as it is apparent from the quality of the joint refinement of 2X0G (Table 3) as well as from the agreement between the back-calculated tensors for the N- and C-terminal domains (Figs. 1 and 2, column “CaM-DAPk protein”). It is worth to note that the refined solution structure is very close to the previously found solution structure 2K61 (see Fig. 3): the distance between the centres of mass of the C-terminal domains of the CaM-DAPk protein structure and of the solution structure decreases from 5.7 to 2.3 Å after the use of rigid body minimization, and the angular deviation is reduced from 33.0° to 15.0°. As a final remark we note that a residual discrepancy is found between the back-calculated tensors of the N-terminal and C-terminal domains, mostly in the magnitude of the tensors and in the metal distance, especially for ytterbium(III). This residual discrepancy can be attributed to a limited but significant mobility as highlighted in (Andrałojć et al. 2014).

#### Structure refinement of CaM in complex with IQ peptide

In the case of CaM bound to IQ peptide, an extensive NMR dataset has been collected by the Griesinger’s group (Russo et al. 2013). The NMR data are in quite good agreement with each of the three monomers in the 2BE6 X-ray structure, but the back-calculated tensor parameters and the residue-specific differences between experimental and back-calculated data highlight some relevant discrepancies. These discrepancies were analysed in the original paper under the assumption that the complex in solution experiences some conformational variability. The experimental data were fit by an ensemble which encompasses the X-ray conformations together with a few selected MD-derived conformations.

We have found that a structural refinement using a single tensor for each metal, i.e.: assuming that the structure is not only rigid but also the same in the crystal and in solution (“F” in Fig. 2), is in relatively good agreement with the data (Table 4). However, some localized residue-specific discrepancies still remain, indicating that the fit is suboptimal. By applying the tensor magnitude constraint and thus allowing the two domains to be differently positioned in solution with respect to the solid state, the quality of the fit improves as well as the agreement between the back-calculated tensors (Fig. 2). The analysis of the residue-specific discrepancies also reveals that most of the biases have been removed (Figs. S3a–S3f). Again, the origin of the slightly different arrangement of the protein domains in solution is likely ascribable to the lack of the intermolecular interactions that connect each peptide-bound CaM with the neighbouring molecules in the crystal (Fig. 4). The small residual differences not accounted for by a single static model were similarly present even considering the NMR data as averaged over multiple X-ray conformations (Russo et al. 2013).

## Conclusions

Only in a limited number of cases the crystal structures and the NMR data are smoothly in agreement with one another, but not all cases of inconsistency are necessarily significant. This is due to the intrinsic short-range inaccuracy of X-ray, especially in determining the position of hydrogen atoms, which are at the core of the NMR observations. Joint refinement of crystal structures against both X-ray experimental data and NMR data has thus proven a powerful tool to detect whether this inconsistency is real or if it is caused by the “structural noise”. To strengthen the use of the NMR data it is useful to impose constraints among the properties of the tensors (magnitude and/or orientation) to reduce the number of unknowns when different tensors need to be used, either because of structural rearrangement between the crystal and the solution or because different samples with differential labelling schemes are used to reduce the spectral complexity in the NMR measurements. We here describe the implementation of such a possibility in REFMAC-NMR, and show the efficiency of this approach in real-life cases. By these examples we demonstrate that using constraints among tensors is beneficial for improving the quality of the refinements even in cases where the experimental data are not sufficient to robustly estimate all the parameters. The changes in the structures are mostly involving heavy atoms and result in an overall improvement of the quality of the structure as evaluated by MolProbity (Chen et al. 2009, see Supplementary Material).

### Electronic supplementary material

Below is the link to the electronic supplementary material.

Supplementary material 1 (DOCX 3299 KB)

## Appendix

### Problem re-parameterization

To allow for the inclusion of the tensor orientation and magnitude constraints, it is convenient to re-express the problem in terms of eigenvectors and eigenvalues of the alignment tensor ***S***. Eigenvectors *e*_*x*_, *e*_*y*_, *e*_*z*_ and associated eigenvalues ${\tilde {S}_{xx}}$, ${\tilde {S}_{yy}}$, ${\tilde {S}_{zz}}$ encode, respectively, the information about the orientation and the magnitude/anisotropy of the tensor ***S***, and are related to the tensor matrix by the simple relationship:

$${\varvec{S}}=R \; A\;{R^T}= \left( {\begin{array}{*{20}{c}} {{{\varvec{e}}_x}}&{{{\varvec{e}}_y}}&{{{\varvec{e}}_z}} \end{array}} \right)\left( {\begin{array}{*{20}{c}} {{{\tilde {S}}_{xx}}}&0&0 \\ 0&{{{\tilde {S}}_{yy}}}&0 \\ 0&0&{{{\tilde {S}}_{zz}}} \end{array}} \right){\left( {\begin{array}{*{20}{c}} {{{\varvec{e}}_x}}&{{{\varvec{e}}_y}}&{{{\varvec{e}}_z}} \end{array}} \right)^T}$$

Eigenvectors basis constitutes the rotation matrix *R* that transforms the tensor from the molecular frame to its principal axis frame. This allows expressing each element ${S_{ij}}$ of the alignment tensor ***S*** in terms of its principal components ${\tilde {S}_{ii}}$ and of element of the rotation matrix *R* as following:

$${\varvec{S}}=R \; A\;{R^T}= \left( {\begin{array}{*{20}{c}} {{r_{11}}}&{{r_{12}}}&{{r_{13}}} \\ {{r_{21}}}&{{r_{22}}}&{{r_{23}}} \\ {{r_{31}}}&{{r_{32}}}&{{r_{33}}} \end{array}} \right)\left( {\begin{array}{*{20}{c}} {{{\tilde {S}}_{xx}}}&0&0 \\ 0&{{{\tilde {S}}_{yy}}}&0 \\ 0&0&{{{\tilde {S}}_{zz}}} \end{array}} \right){\left( {\begin{array}{*{20}{c}} {{r_{11}}}&{{r_{12}}}&{{r_{13}}} \\ {{r_{21}}}&{{r_{22}}}&{{r_{23}}} \\ {{r_{31}}}&{{r_{32}}}&{{r_{33}}} \end{array}} \right)^T}$$

$${S_{ij}}=\mathop \sum \limits_{{k=1}}^{3} {r_{ik}}{\tilde {S}_{kk}}{r_{kj}}.$$

Each element of the rotation matrix *R* is then more conveniently expressed in terms of quaternions, thus reducing the number of parameters describing the rotation from nine to four.

Given *f* the target function to be minimized, *x* the set of parameters for the original problem, defined as:

$$x=\left[ {{{\text{S}}_{zz}},{{\text{S}}_{xx}} - {{\text{S}}_{yy}},{S_{xy}},{S_{xz}},{S_{yz}}} \right].$$

and *p* the set of parameters for the re-parameterized problem, defined in this particular case as:

$$p=\left[ {{q_0}, {q_1}, {q_2}, {q_3},{{\tilde {S}}_{xx}}, {{\tilde {S}}_{yy}}} \right]$$

the first and second derivatives of the re-parameterized problem can then be directly calculated starting from the derivatives of the original problem by application of the chain rule, as following:

$$\frac{{\partial f}}{{\partial {p_j}}}=\mathop \sum \limits_{i} \frac{{\partial f}}{{\partial {x_i}}}\frac{{\partial {x_i}}}{{\partial {p_j}}}$$

$$\frac{{{\partial ^2}f}}{{\partial {p_i}\partial {p_j}}}=\mathop \sum \limits_{{k,l}} \frac{{\partial {x_k}}}{{\partial {p_i}}}\frac{{{\partial ^2}f}}{{\partial {x_k}\partial {x_l}}}\frac{{\partial {x_k}}}{{\partial {p_i}}}+\mathop \sum \limits_{k} \frac{{\partial f}}{{\partial {x_k}}}\frac{{{\partial ^2}{x_k}}}{{\partial {p_i}\partial {p_j}}}$$

where the last term of the second derivative has not be considered since it implies the use of the first derivative with the respect of the original problem, making the optimization less stable.

### Imposing anisotropy and orientation constraints between tensors

In the presence of two structural units, two independent tensors can be defined, ***S***_*a*_ and ***S***_*b*_, depending on the two sets of the parameters *p*_*a*_ and *p*_*b*_ defined as:

$${p_a}=\left[ {{q_{a,i}},{{\tilde {S}}_{a,ii}}} \right],{p_b}=\left[ {{q_{b,i}},{{\tilde {S}}_{b,ii}}} \right]\;\text{with}\; i = 0, 1, 2, 3 \;\text{and}\;ii = xx, yy$$

The function *f* is thus the sum of the functions referring to each domain:

$$f\left( {p^{\prime}} \right)=f\left( {{p_a},{p_b}} \right)={f_a}\left( {{p_a}} \right)+{f_b}\left( {{p_b}} \right)={f_a}\left( {{q_{a,i}},{{\tilde {S}}_{a,ii}}} \right)+{f_b}\left( {{q_{b,i}},{{\tilde {S}}_{b,ii}}} \right)$$

where

$$p^{\prime}=\left[ {{p_a},{p_b}} \right]=\left[ {{q_{a,i}},{{\tilde {S}}_{a,ii}},{q_{b,i}},{{\tilde {S}}_{b,ii}}} \right]$$

The first and second derivatives of *f* are given by the derivatives of *f*_*a*_ with respect to the *p*_*a*_ parameters and of *f*_*b*_ with respect to the *p*_*b*_ parameters. The derivatives are reported, for simplicity, in the following matrix form where only the non-null elements are shown:

$$\frac{{\partial f}}{{\partial p^{\prime}}}=\left[ {\frac{{\partial {f_a}}}{{\partial {q_{a,i}}}}; \;\frac{{\partial {f_a}}}{{\partial {{\tilde {S}}_{a,ii}}}};\frac{{\partial {f_b}}}{{\partial {q_{b,i}}}};\;\frac{{\partial {f_b}}}{{\partial {{\tilde {S}}_{b,ii}}}}} \right]$$

$$\frac{{{\partial ^2}f}}{{\partial {{p^{\prime}}^2}}}=\left[ {\begin{array}{*{20}{c}} {\begin{array}{*{20}{c}} {\frac{{{\partial ^2}{f_a}}}{{\partial {q_{a,i}}\partial {q_{a,j}}}}}&{\frac{{{\partial ^2}{f_a}}}{{\partial {q_{a,i}}\partial {{\tilde {S}}_{a,jj}}}}} \\ {\frac{{{\partial ^2}{f_a}}}{{\partial {q_{a,i}}\partial {{\tilde {S}}_{a,jj}}}}}&{\frac{{{\partial ^2}{f_a}}}{{\partial {{\tilde {S}}_{a,ii}}\partial {{\tilde {S}}_{a,jj}}}}} \end{array}}&{} \\ {}&{\begin{array}{*{20}{c}} {\frac{{{\partial ^2}{f_b}}}{{\partial {q_{b,i}}\partial {q_{b,j}}}}}&{\frac{{{\partial ^2}{f_b}}}{{\partial {q_{b,i}}\partial {{\tilde {S}}_{b,jj}}}}} \\ {\frac{{{\partial ^2}{f_b}}}{{\partial {q_{b,i}}\partial {{\tilde {S}}_{b,jj}}}}}&{\frac{{{\partial ^2}{f_b}}}{{\partial {{\tilde {S}}_{b,ii}}\partial {{\tilde {S}}_{b,jj}}}}} \end{array}} \end{array}} \right]$$

In order to constrain the orientation of the two tensors to be the same (${q_{a,i}}={q_{b,i}}\,for\,i\,=\,0,{\text{ }}1,{\text{ }}2,{\text{ }}3$), parameters and target function are expressed in the following way:

$$p^{\prime}=\left[ {{q_i},{{\tilde {S}}_{a,ii}},{{\tilde {S}}_{b,ii}}} \right] \quad f\left( {p^{\prime}} \right)={f_a}\left( {{q_i},{{\tilde {S}}_{a,ii}}} \right)+{f_b}\left( {{q_i},{{\tilde {S}}_{b,ii}}} \right)$$

where the non-null elements of the first and second derivatives of *f* are given as following:

$$\frac{{\partial f}}{{\partial {p^{\prime}}}}=\left[ {\frac{{\partial {f_a}}}{{\partial {q_i}}}+\frac{{\partial {f_b}}}{{\partial {q_i}}}; \;\frac{{\partial {f_a}}}{{\partial {{\mathop S\limits^{} }_{a,ii}}}};\;\frac{{\partial {f_b}}}{{\partial {{\mathop S\limits^{} }_{b,ii}}}}} \right]$$

$$\frac{{{\partial ^2}f}}{{\partial {p^{'2}}}}=\left[ {\begin{array}{*{20}{c}} {\frac{{{\partial ^2}{f_a}}}{{\partial {q_i}\partial {q_j}}}+\frac{{{\partial ^2}{f_b}}}{{\partial {q_i}\partial {q_j}}}}&{\frac{{{\partial ^2}{f_a}}}{{\partial {q_i}\partial {{\tilde {S}}_{a,jj}}}}}&{\frac{{{\partial ^2}{f_b}}}{{\partial {q_i}\partial {{\tilde {S}}_{b,jj}}}}} \\ {\frac{{{\partial ^2}{f_a}}}{{\partial {q_i}\partial {{\tilde {S}}_{a,jj}}}}}&{\frac{{{\partial ^2}{f_a}}}{{\partial {{\tilde {S}}_{a,ii}}\partial {{\tilde {S}}_{a,jj}}}}}&{} \\ {\frac{{{\partial ^2}{f_b}}}{{\partial {q_i}\partial {{\tilde {S}}_{b,jj}}}}}&{}&{\frac{{{\partial ^2}{f_b}}}{{\partial {{\tilde {S}}_{b,ii}}\partial {{\tilde {S}}_{b,jj}}}}} \end{array}} \right]$$

In order to constrain the anisotropy values of the two tensors to be the same (${\tilde {S}_{a,ii}}={\tilde {S}_{b,ii}}$ for *ii* = *xx, yy*), parameters and target function can be re-expressed in the following way:

$$p^{\prime}=\left[ {{q_{a,i}},{{\tilde {S}}_{ii}},{q_{b,i}}} \right]\quad f\left( {p^{\prime}} \right)={f_a}\left( {{q_{a,i}},{{\tilde {S}}_{ii}}} \right)+{f_b}\left( {{q_{b,i}},{{\tilde {S}}_{ii}}} \right)$$

where the non-null elements of the first and second derivatives of *f* are given as following:

$$\frac{{\partial f}}{{\partial p^{\prime}}}=\left[ {\frac{{\partial {f_a}}}{{\partial {q_{a,i}}}};\;\frac{{\partial {f_a}}}{{\partial {{\tilde {S}}_{ii}}}}+\frac{{\partial {f_b}}}{{\partial {{\tilde {S}}_{ii}}}};\frac{{\partial {f_b}}}{{\partial {q_{b,i}}}}} \right]$$

$$\frac{{{\partial ^2}f}}{{\partial {p^{\prime 2}}}}=\left[ {\begin{array}{*{20}{c}} {\frac{{{\partial ^2}{f_a}}}{{\partial {q_{a,i}}\partial {q_{a,j}}}}}&{\frac{{{\partial ^2}{f_a}}}{{\partial {q_{a,i}}\partial {{\tilde {S}}_{jj}}}}}&{} \\ {\frac{{{\partial ^2}{f_a}}}{{\partial {q_{a,i}}\partial {{\tilde {S}}_{jj}}}}}&{\frac{{{\partial ^2}{f_a}}}{{\partial {{\tilde {S}}_{ii}}\partial {{\tilde {S}}_{jj}}}}+\frac{{{\partial ^2}{f_b}}}{{\partial {{\tilde {S}}_{ii}}\partial {{\tilde {S}}_{jj}}}}}&{\frac{{{\partial ^2}{f_b}}}{{\partial {q_{b,i}}\partial {{\tilde {S}}_{jj}}}}} \\ {\frac{{{\partial ^2}{f_b}}}{{\partial {q_{b,i}}\partial {{\tilde {S}}_{jj}}}}}&{\frac{{{\partial ^2}{f_b}}}{{\partial {q_{b,i}}\partial {q_{b,j}}}}}&{} \end{array}} \right]$$

This approach can be used to perform the minimization of a generic number of tensors, constrained to each other by their anisotropy, orientation or both.
