# Correction to: Joint X-ray/NMR structure refinement of multidomain/multisubunit systems

**DOI:** 10.1007/s10858-019-00238-4

**Published:** 2019-05-08

**Authors:** Azzurra Carlon, Enrico Ravera, Giacomo Parigi, Garib N. Murshudov, Claudio Luchinat

**Affiliations:** 1 Magnetic Resonance Center (CERM) and Interuniversity Consortium for Magnetic Resonance of Metallo Proteins (CIRMMP), Via L. Sacconi 6, 50019 Sesto Fiorentino, Italy; 2 Department of Chemistry “Ugo Schiff”, University of Florence, Via della Lastruccia 3, 50019 Sesto Fiorentino, Italy; 3 MRC Laboratory for Molecular Biology, Francis Crick Ave, CB2 0QH Cambridge, UK

## Correction to: Journal of Biomolecular NMR 10.1007/s10858-018-0212-3

The article “Joint X-ray/NMR structure refinement of multidomain/multisubunit systems” written by “Azzurra Carlon, Enrico Ravera, Giacomo Parigi, Garib N. Murshudov and Claudio Luchinat” was originally published electronically on the publisher’s internet portal (currently SpringerLink) on 11 October 2018 without open access.

With the author(s)’ decision to opt for Open Choice the copyright of the article changed on 7 May 2019 to © The Author(s) 2019 and the article is forthwith distributed under the terms of the Creative Commons Attribution 4.0 International License (http://creativecommons.org/licenses/by/4.0/), which permits use, duplication, adaptation, distribution and reproduction in any medium or format, as long as you give appropriate credit to the original author(s) and the source, provide a link to the Creative Commons license and indicate if changes were made. The original article has been corrected.

