# Supplementary material for: Joint X-ray/NMR structure refinement of multidomain/multisubunit systems
Source: J Biomol NMR. 2018 Oct 11;73(6):265–78. doi: 10.1007/s10858-018-0212-3 (PMC6692505; doi:10.1007/s10858-018-0212-3)
Supplement: Supplementary file 1 — Supplementary material 1 (DOCX 3299 KB) [file 10858_2018_212_MOESM1_ESM.docx]

**Joint X-ray/NMR structure refinement of multidomain/multisubunit systems**

Azzurra Carlon,^1^ Enrico Ravera,^1,2^ Giacomo Parigi,^1,2^ Garib N. Murshudov,^3,^* Claudio Luchinat^1,2^*

1. Magnetic Resonance Center (CERM) and Interuniversity Consortium for Magnetic Resonance of Metallo Proteins (CIRMMP), Via L. Sacconi 6, 50019 Sesto Fiorentino, Italy,
2. Department of Chemistry “Ugo Schiff”, University of Florence, Via della Lastruccia 3, 50019 Sesto Fiorentino, Italy,
3. MRC Laboratory for Molecular Biology, Francis Crick Ave, Cambridge, CB2 0QH, UK

**SUPPLEMENTARY MATERIAL**

**Table S1:** Comparison of the tensors calculated independently for refined structures of Sxl and CSD1 before and after the inclusion of orientation constraints, in terms of tensor magnitude and alignment. Q-factors for the individual Sxl and CSD1 domains are also reported

| Constraint | | | |
| --- | --- | --- | --- |
|  | Q-factor RDCs  $Q_{\mathrm{Sxl}}, Q_{CSD1}$ | Tensor magnitude  $\frac{A_{\mathrm{Sxl}}}{A_{CSD1}}$ | Tensor alignment  $\frac{S_{\mathrm{Sxl}}\cdot S_{CSD1}}{\left\Vert S_{\mathrm{Sxl}} \right\Vert\left\Vert S_{CSD1} \right\Vert}$ |
| Chains A, X | 0.151, 0.071 | 0.751 | 0.997 |
| Chains B, Y | 0.143, 0.074 | 0.753 | 0.996 |
| Scaled tensor | | | |
|  | Q-factor RDCs  $Q_{\mathrm{Sxl}}, Q_{CSD1}$ | Tensor magnitude  $\frac{A_{\mathrm{Sxl}}}{A_{CSD1}}$ | Tensor alignment  $\frac{S_{\mathrm{Sxl}}\cdot S_{CSD1}}{\left\Vert S_{\mathrm{Sxl}} \right\Vert\left\Vert S_{CSD1} \right\Vert}$ |
| Chains A, X | 0.150, 0.090 | 0.777 | 0.997 |
| Chains B, Y | 0.139, 0.088 | 0.791 | 0.998 |

**Table S2.** Comparison of the MolProbity analysis results between the structures refined with REFMAC and with REFMAC-NMR. The Q-factors are recalculated after rebuilding the hydrogen atoms using REDUCE. This confirms that the NMR refinement is mostly affecting the coordinates of the heavy atoms.

| PDB code: 4QQB – Resolution: 2.80 Å | | | | | |
| --- | --- | --- | --- | --- | --- |
| Parameters | Goal | REFMAC 5.9.0000 refinement  Reduce applied | | REFMAC 5.9.0000 refinement +NMR  Reduce applied | |
| **Clashscore, all atoms** |  | **6.96** | **99th percentile (N=141, 2.80Å ± 0.25Å)** | **4.45** | **100th percentile (N=141, 2.80Å ± 0.25Å)** |
| Poor rotamers | <0.3% | 37 | 9.89% | 36 | 8.65% |
| Favored rotamers | >98% | 326 | 78.37% | 327 | 78.61% |
| Ramachandran outliers | <0.05% | 5 | 1.05% | 5 | 1.05% |
| Ramachandran favored | >98% | 438 | 92.41% | 435 | 91.77% |
| **MolProbity score** |  | **2.58** | **88th percentile (N=4482, 2.80Å ± 0.25Å)** | **2.43** | **93rd percentile (N=4482, 2.80Å ± 0.25Å)** |
| Cβ deviations > 0.25 Å | Goal: 0 | 0 | 0% | 0 | 0% |
| Bad bonds | Goal: 0% | 0/3906 | 0% | 5/3906 | 0.13% |
| Bad angles | Goal: <0.1% | 0/5262 | 0% | 2/5262 | 0.04% |
| Cis Prolines | Expected: ≤1 per chain, or ≤5% | 0/18 | 0% | 0/18 | 0% |
| Probably wrong sugar puckers | Goal: 0 | 2 | 5.88% | 2 | 5.88% |
| Bad backbone conformations | Goal: <= 5% | 15 | 44.12% | 15 | 44.12% |
| Bad bonds | Goal: 0% | 0/796 | 0% | 0/796 | 0.00% |
| Bad angles | Goal: <0.1% | 13/1234 | 1.05% | 14/1234 | 1.13% |
| CaBLAM outliers | Goal: <1.0% | 14 | 3.00% | 11 | 2.36% |
| CA Geometry outliers | Goal: <0.5% | 2 | 0.43% | 2 | 0.43% |
| **Q-factor RDC** | after REFMAC-NMR refinement: **0.121** | **0.428** | | **0.132** | |

| PDB code: 2X0G – Resolution: 2.20 Å | | | | | |
| --- | --- | --- | --- | --- | --- |
| Parameters | Goal | REFMAC 5.9.0000 refinement  Reduce applied | | REFMAC 5.9.0000 refinement +NMR  Reduce applied | |
| **Clashscore, all atoms** |  | **4.84** | **98th percentile (N=456, 2.20Å ± 0.25Å)** | **4.44** | **98th percentile (N=456, 2.20Å ± 0.25Å)** |
| Poor rotamers | <0.3% | 22 | 5.56% | 20 | 5.05% |
| Favored rotamers | >98% | 355 | 89.65% | 354 | 89.39% |
| Ramachandran outliers | <0.05% | 1 | 0.23% | 1 | 0.23% |
| Ramachandran favored | >98% | 433 | 97.52% | 432 | 97.30% |
| **MolProbity score** |  | **1.95** | **88th percentile (N=10167, 2.20Å ± 0.25Å)** | **1.95** | **88th percentile (N=10167, 2.20Å ± 0.25Å)** |
| Cβ deviations > 0.25 Å | 0 | 0 | 0% | 1 | 0.23% |
| Bad bonds | 0% | 1/3700 | 0.03% | 3/3700 | 0.08% |
| Bad angles | <0.1% | 5/4984 | 0.1% | 8/4984 | 0.16% |
| Cis Prolines | Expected: ≤1 per chain, or ≤5% | 0/13 | 0% | 0/13 | 0% |
| **Q-factor PCS** | after REFMAC-NMR refinement: **0.098** | **0.191** | | **0.098** | |
| **Q-factor RDC** | after REFMAC-NMR refinement: **0.149** | **0.546** | | **0.166** | |

| PDB code: 2BE6 – Resolution: 2.00 Å | | | | | |
| --- | --- | --- | --- | --- | --- |
| Parameters | Goal | REFMAC 5.9.0000 refinement  Reduce applied | | REFMAC 5.9.0000 refinement +NMR  Reduce applied | |
| **Clashscore, all atoms** |  | **15.24** | **56th percentile (N=715, 2.00Å ± 0.25Å)** | **12.57** | **70th percentile (N=715, 2.00Å ± 0.25Å)** |
| Poor rotamers | <0.3% | 32 | 7.94% | 30 | 7.44% |
| Favored rotamers | >98% | 348 | 86.35% | 348 | 86.35% |
| Ramachandran outliers | <0.05% | 0 | 0 % | 0 | 0% |
| Ramachandran favored | >98% | 458 | 99.13% | 457 | 98.92% |
| **MolProbity score** |  | **2.37** | **44th percentile (N=12522, 2.00Å ± 0.25Å)** | **2.27** | **52th percentile (N=12522, 2.00Å ± 0.25Å)** |
| Cβ deviations > 0.25 Å | 0 | 0 | 0% | 0 | 0% |
| Bad bonds | 0% | 0/3901 | 0.03% | 5/3901 | 0.13% |
| Bad angles | <0.1% | 10/5243 | 0.1% | 16/5243 | 0.31% |
| Cis Prolines | Expected: ≤1 per chain, or ≤5% | 0/6 | 0% | 0/6 | 0% |
| **Q-factor PCS** | after REFMAC-NMR refinement: **0.067** | **0.154** | | **0.067** | |
| **Q-factor RDC** | after REFMAC-NMR refinement: **0.098** | **0.273** | | **0.113** | |


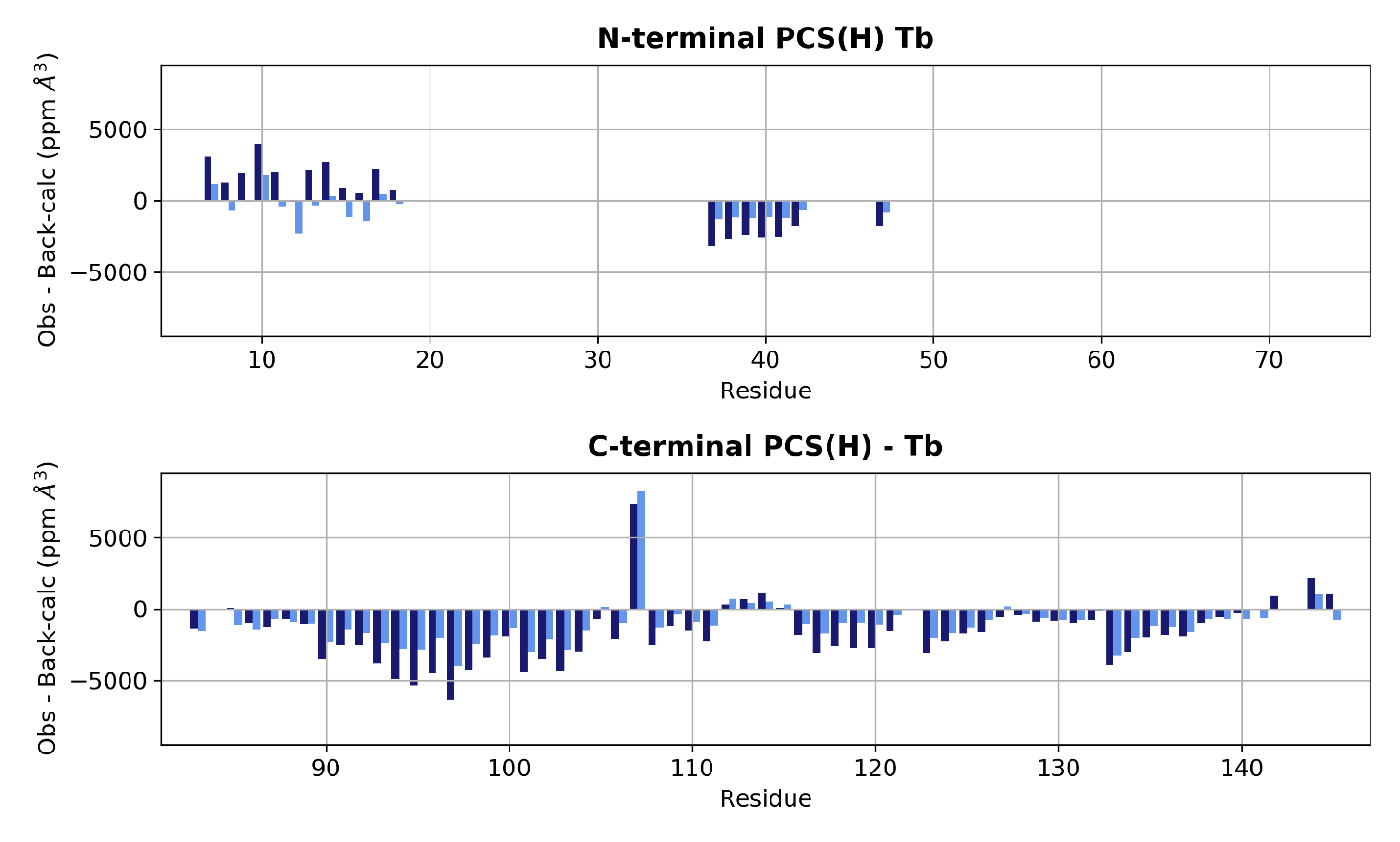


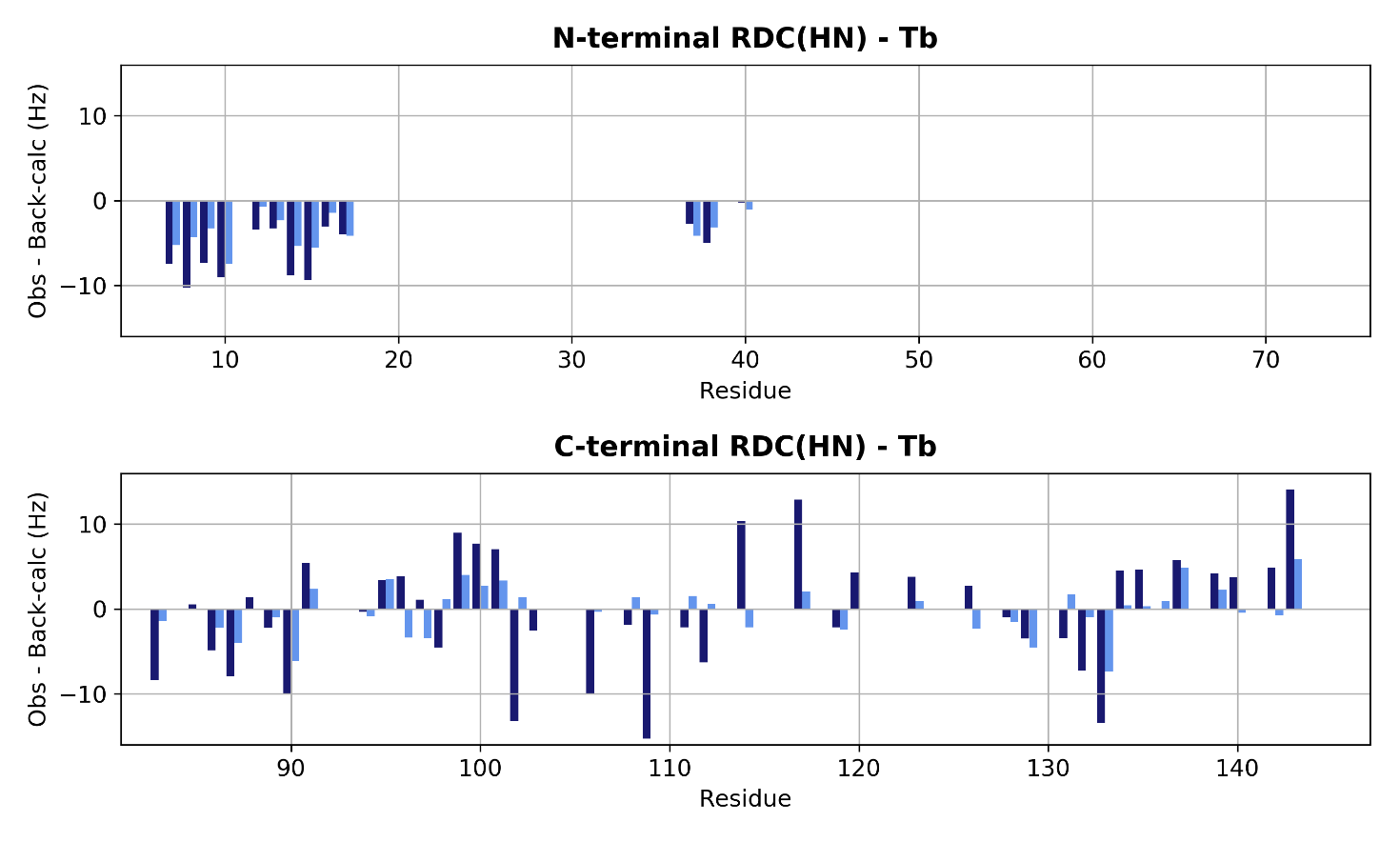


**Figure S1a**: residue-by residue discrepancies calculated for the CaM-DAPk peptide (1YR5) for Tb(III) for the structures refined by REFMAC (without NMR) (dark blue) and for the structures refined by REFMAC-NMR using the tensor magnitude constraint after applying rigid body minimization (light blue).


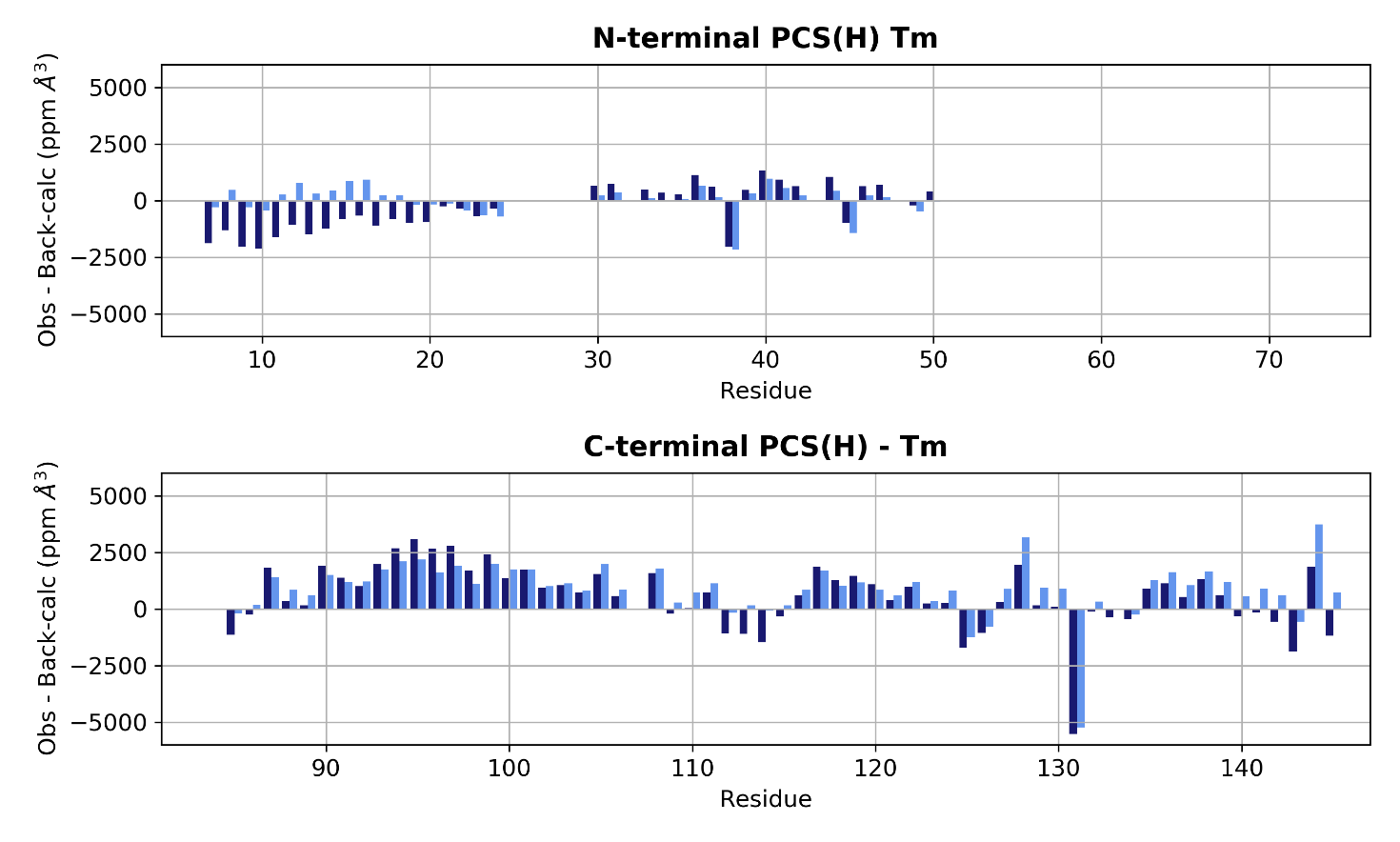


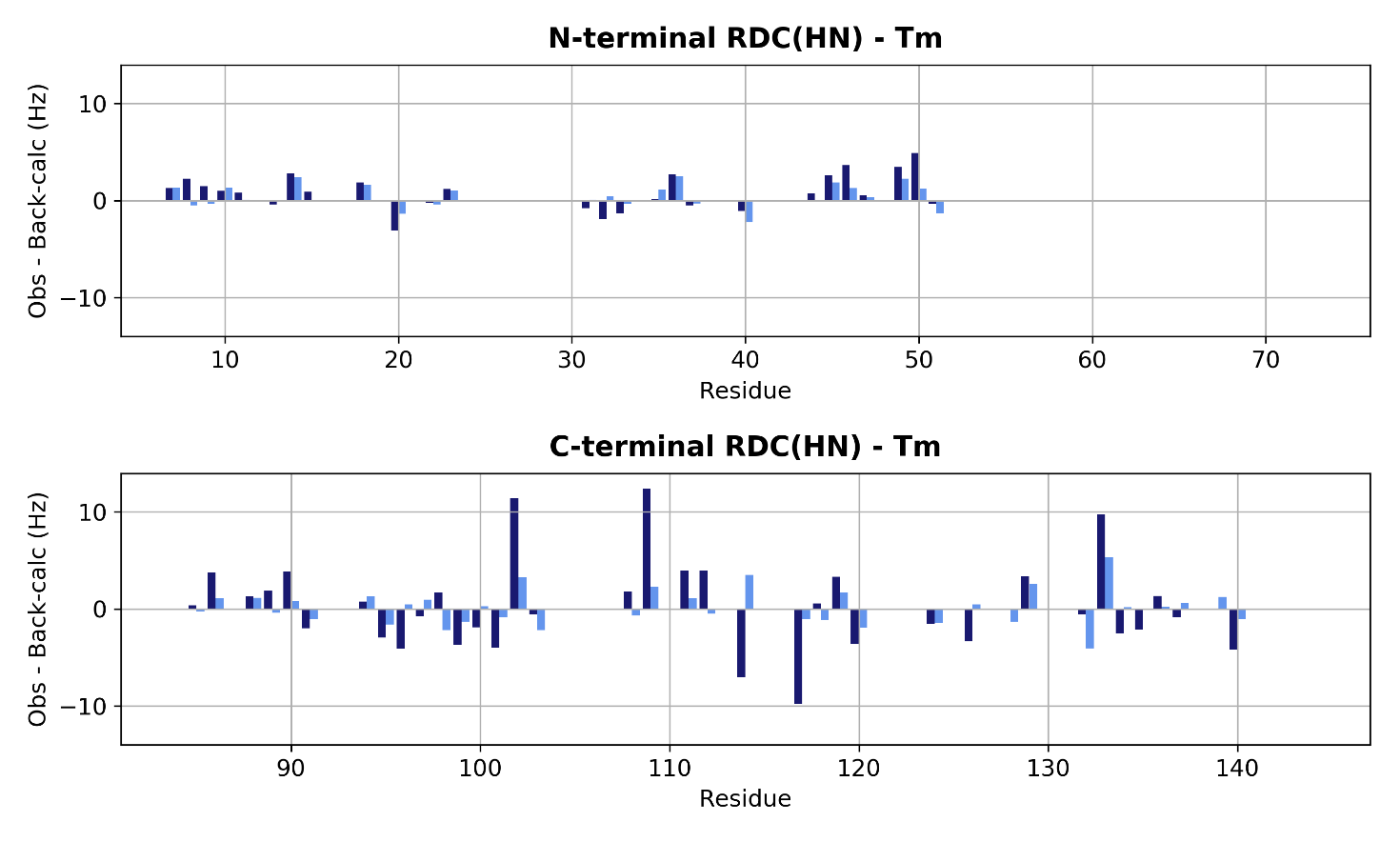


**Figure S1b**: residue-by residue discrepancies calculated for the CaM-DAPk peptide (1YR5) for Tm(III) for the structures refined by REFMAC (without NMR) (dark blue) and for the structures refined by REFMAC-NMR using the tensor magnitude constraint after applying rigid body minimization (light blue).


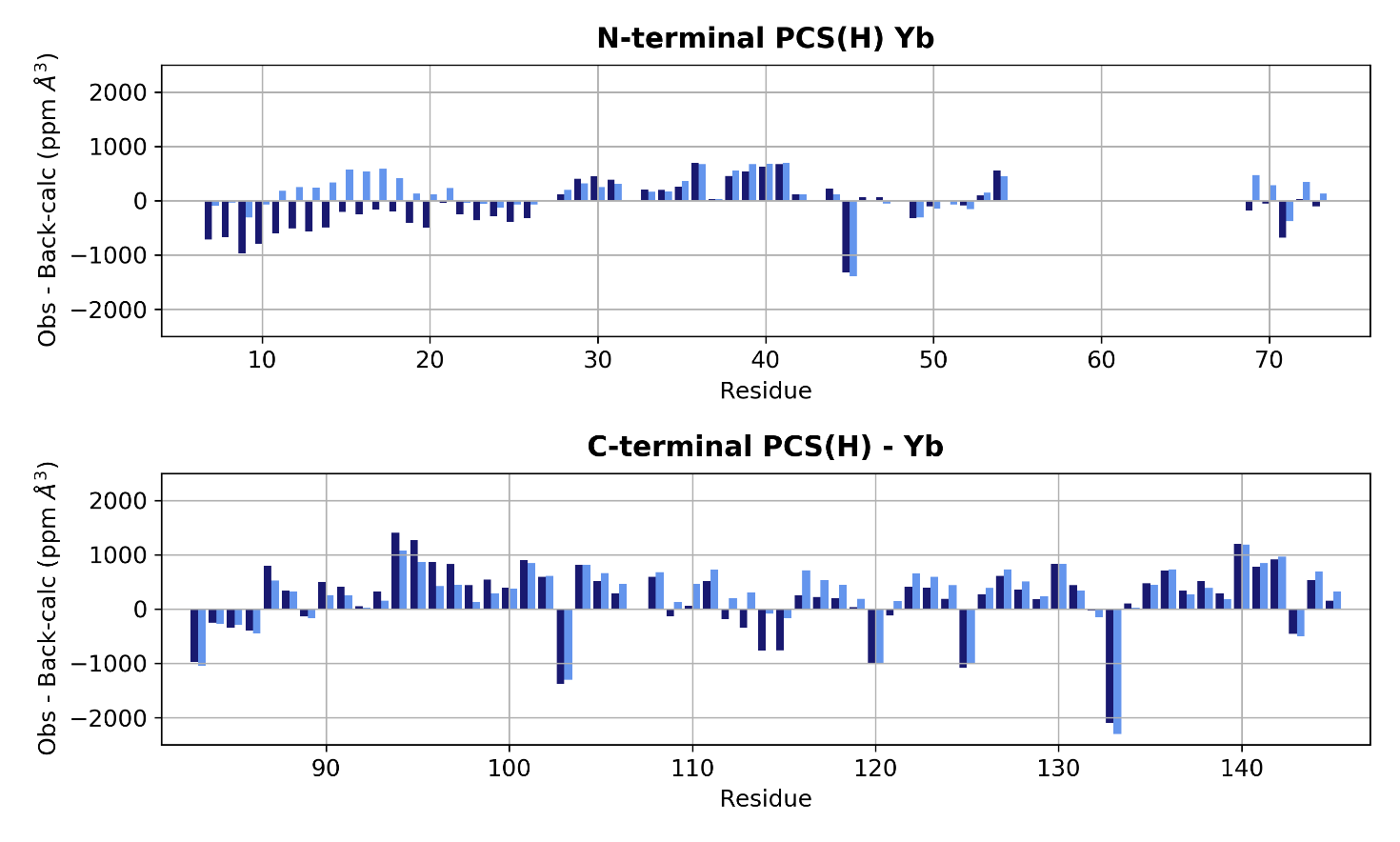


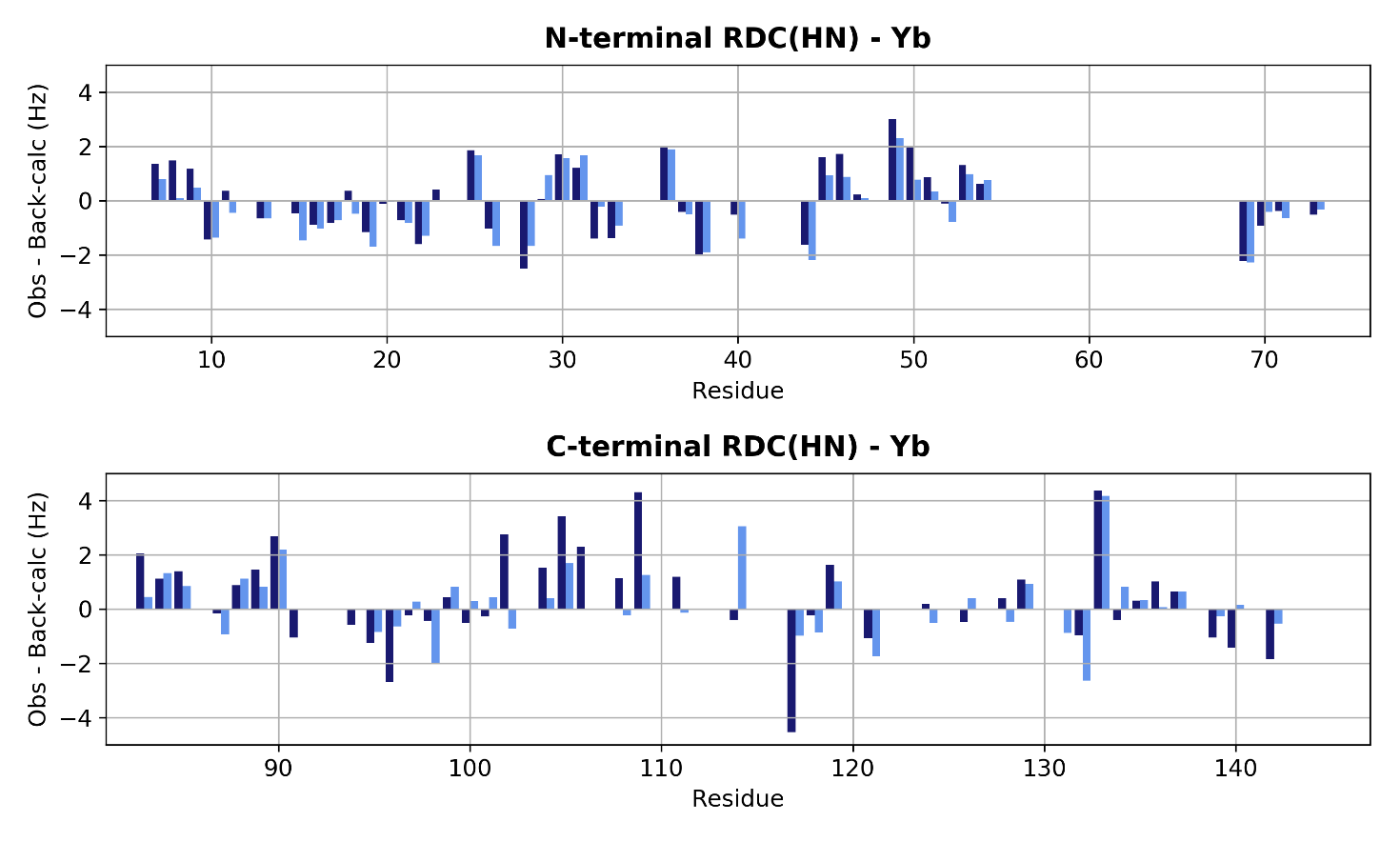
**Figure S1c**: residue-by residue discrepancies calculated for the CaM-DAPk peptide (1YR5) for Yb(III) for the structures refined by REFMAC (without NMR) (dark blue) and for the structures refined by REFMAC-NMR using the tensor magnitude constraint after applying rigid body minimization (light blue).


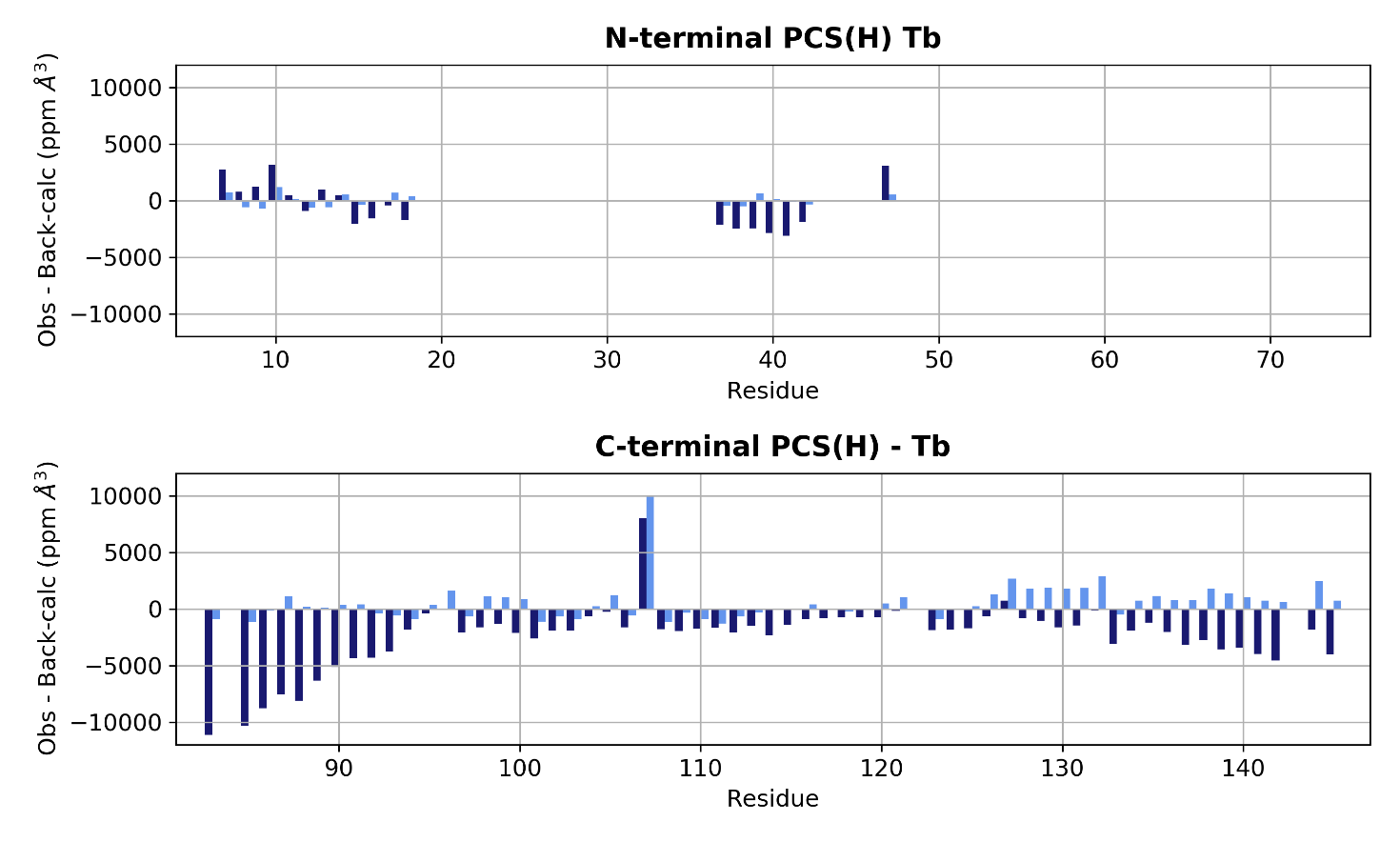


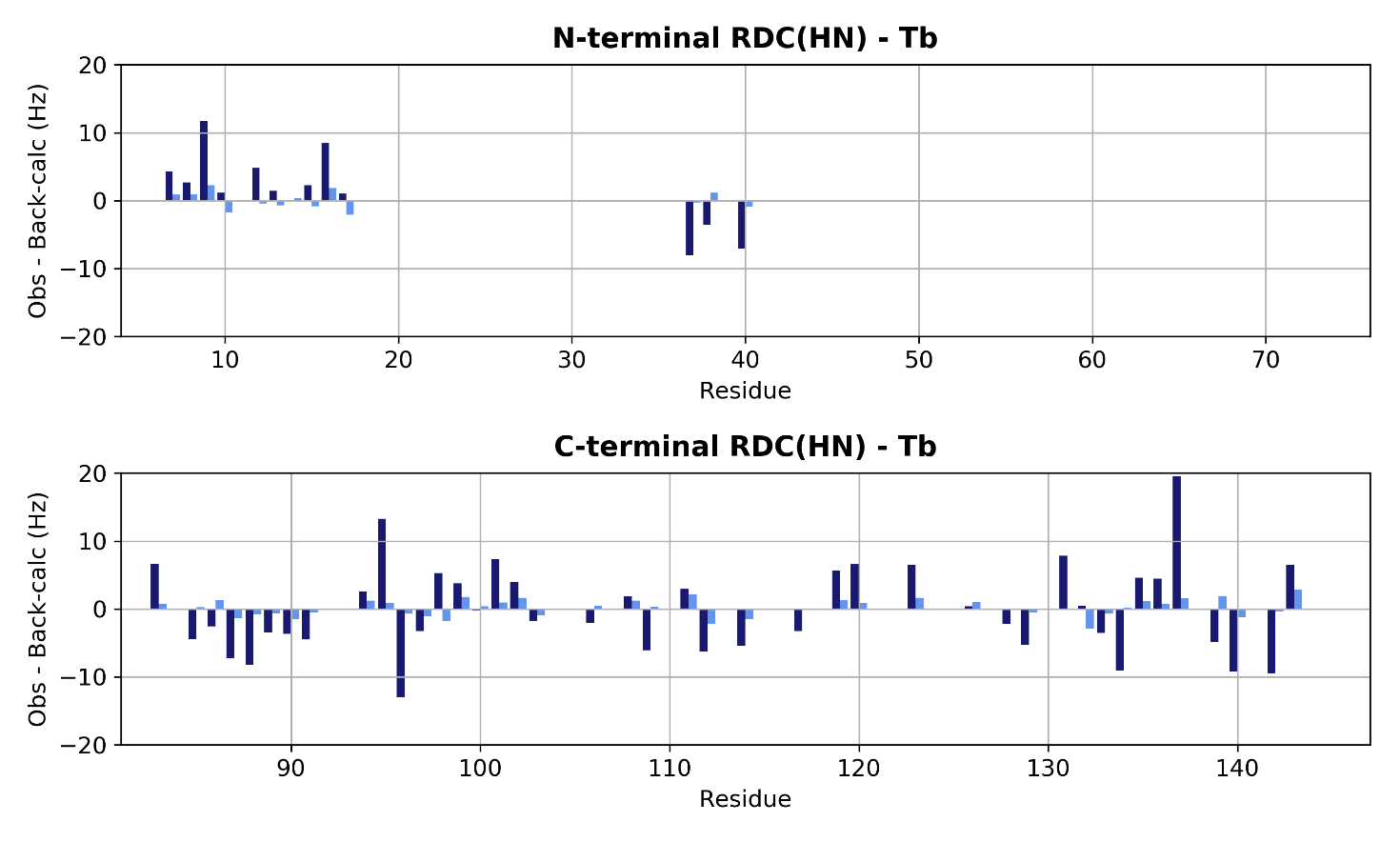


**Figure S2a**: residue-by residue discrepancies calculated for the CaM-DAPk protein (2X0G) for Tb(III) for the structures refined by REFMAC (without NMR) (dark blue) and for the structures refined by REFMAC-NMR using the tensor magnitude constraint after applying rigid body minimization (light blue).


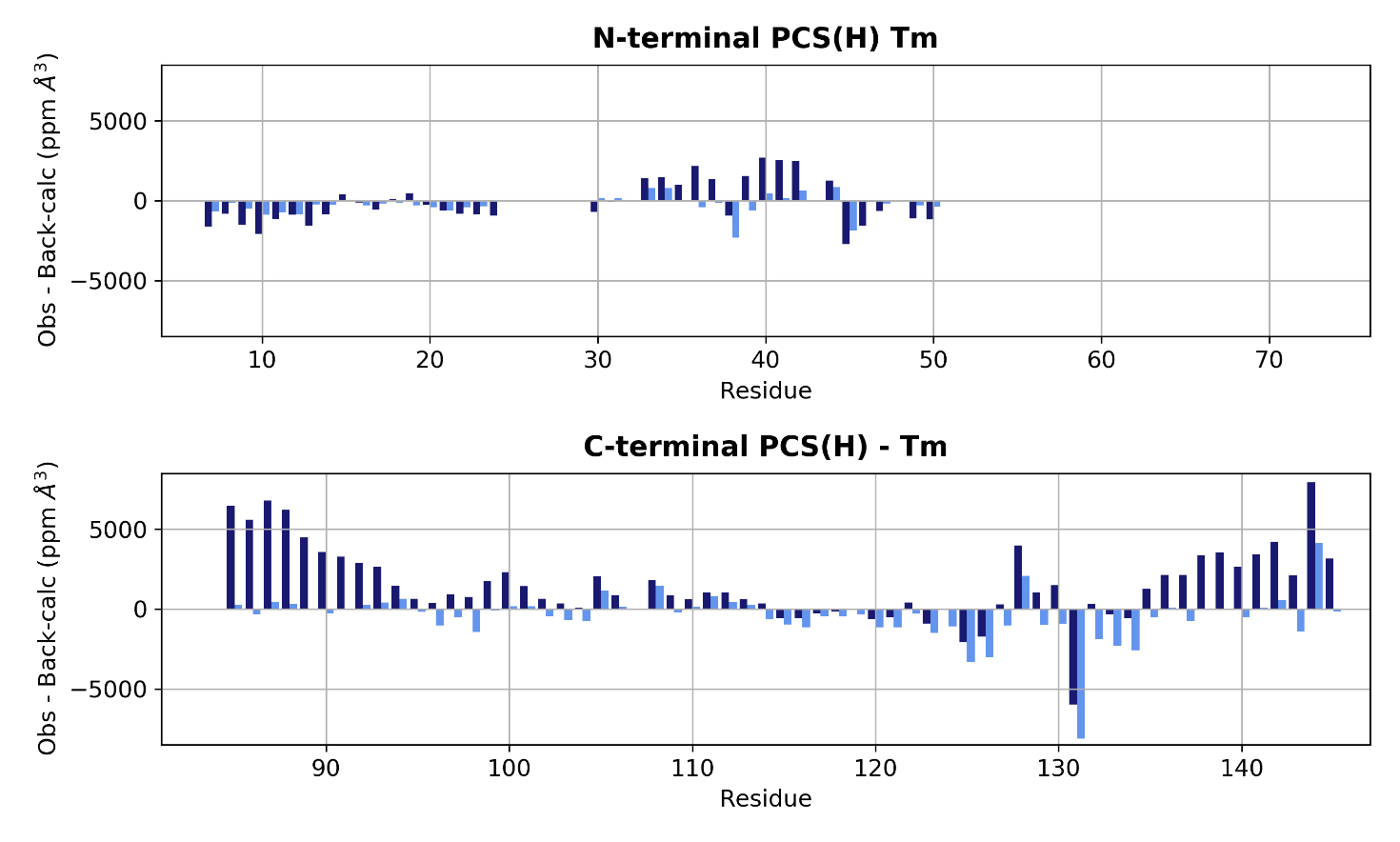


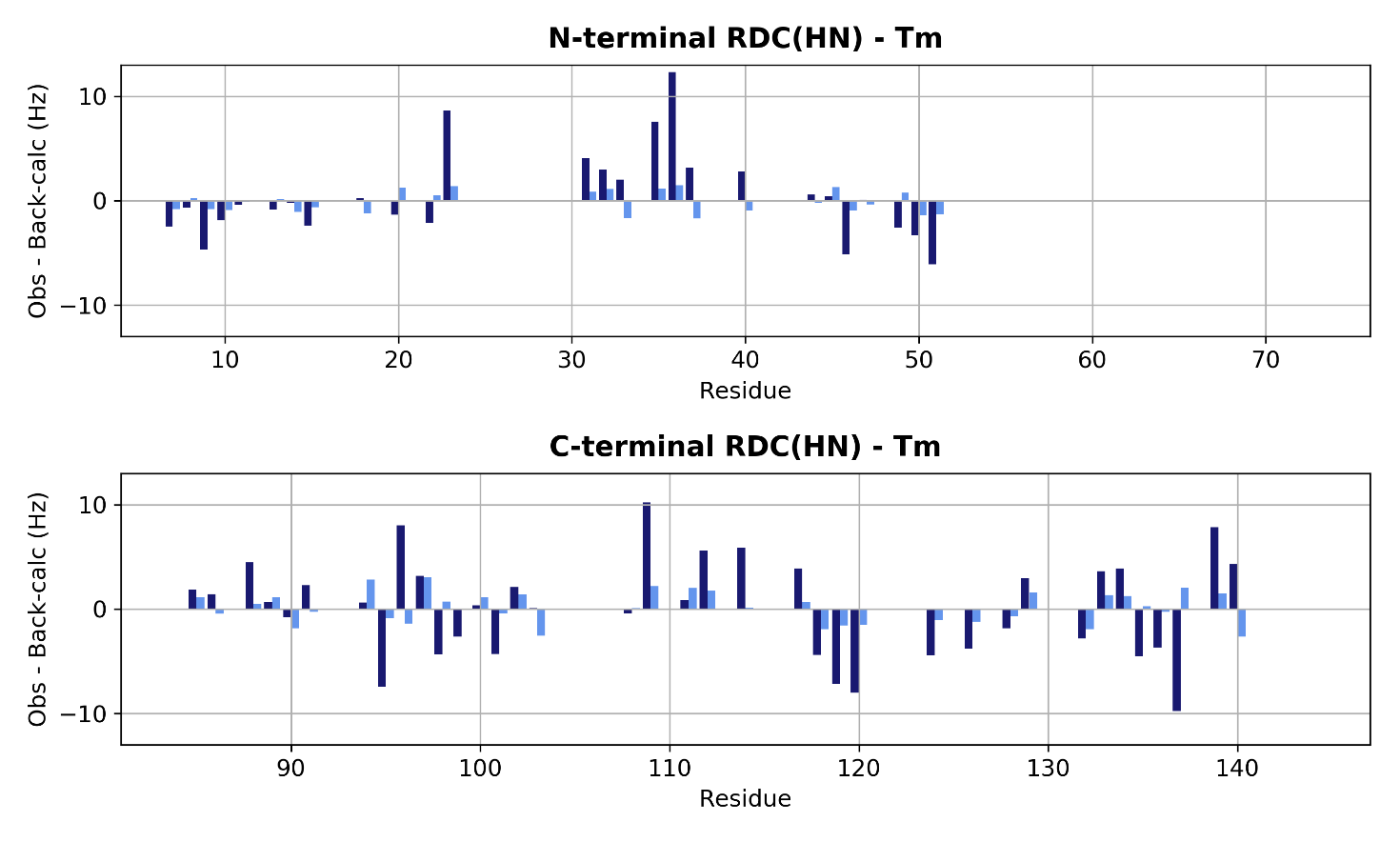


**Figure S2b**: residue-by residue discrepancies calculated for the CaM-DAPk protein (2X0G) for Tm(III) for the structures refined by REFMAC (without NMR) (dark blue) and for the structures refined by REFMAC-NMR using the tensor magnitude constraint after applying rigid body minimization (light blue).


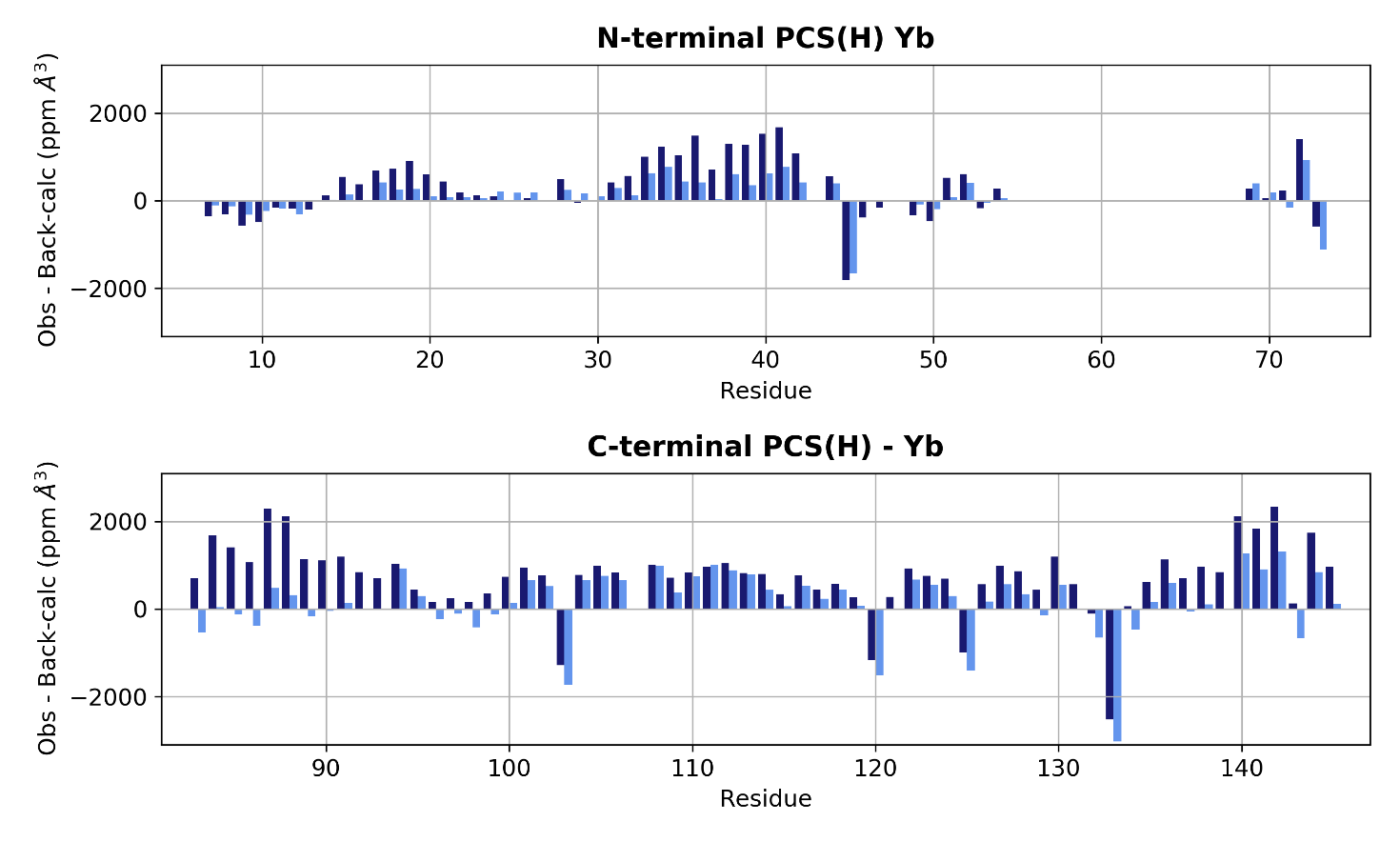


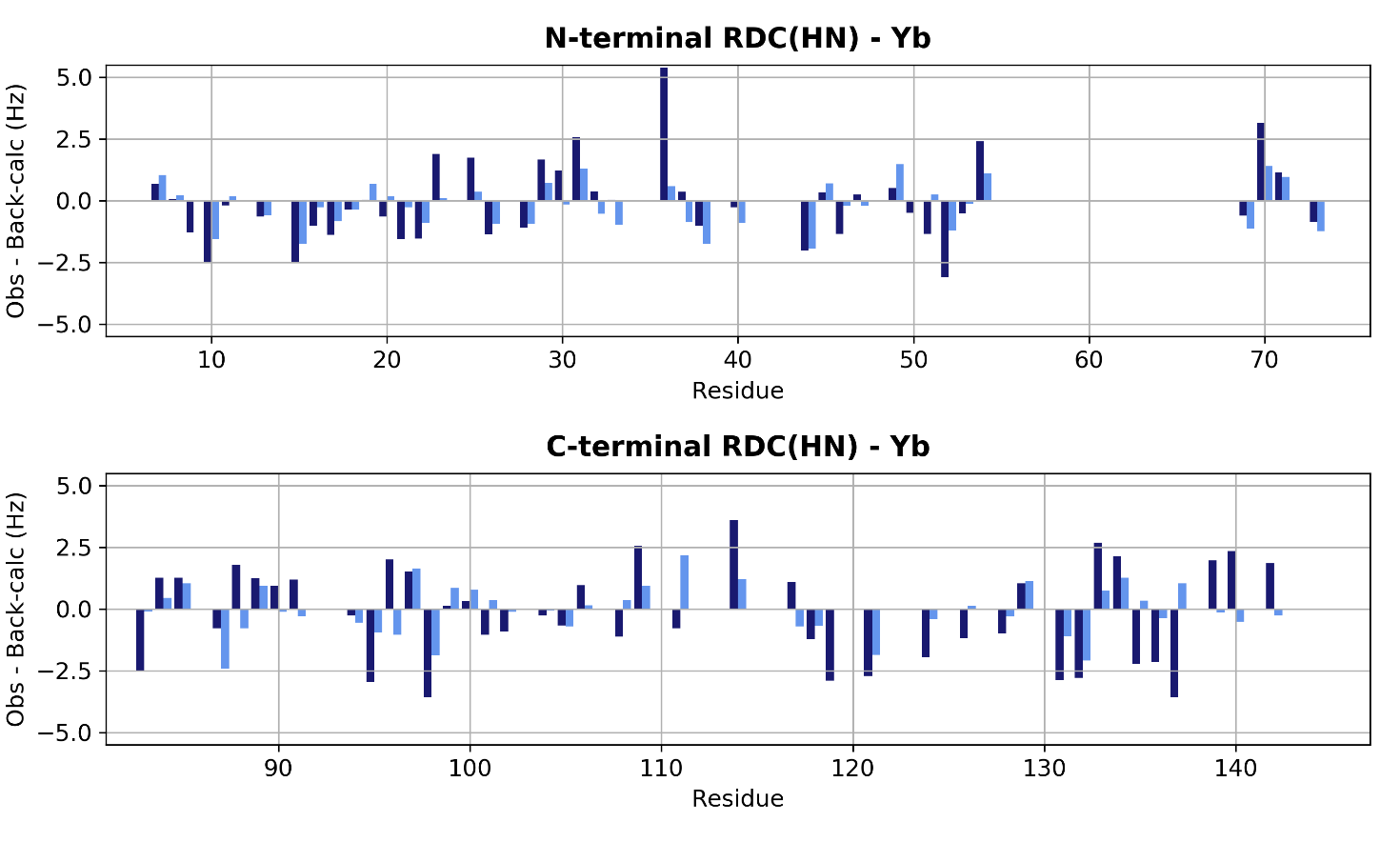


**Figure S2c**: residue-by residue discrepancies calculated for the CaM-DAPk protein (2X0G) for Yb(III) for the structures refined by REFMAC (without NMR) (dark blue) and for the structures refined by REFMAC-NMR using the tensor magnitude constraint after applying rigid body minimization (light blue).


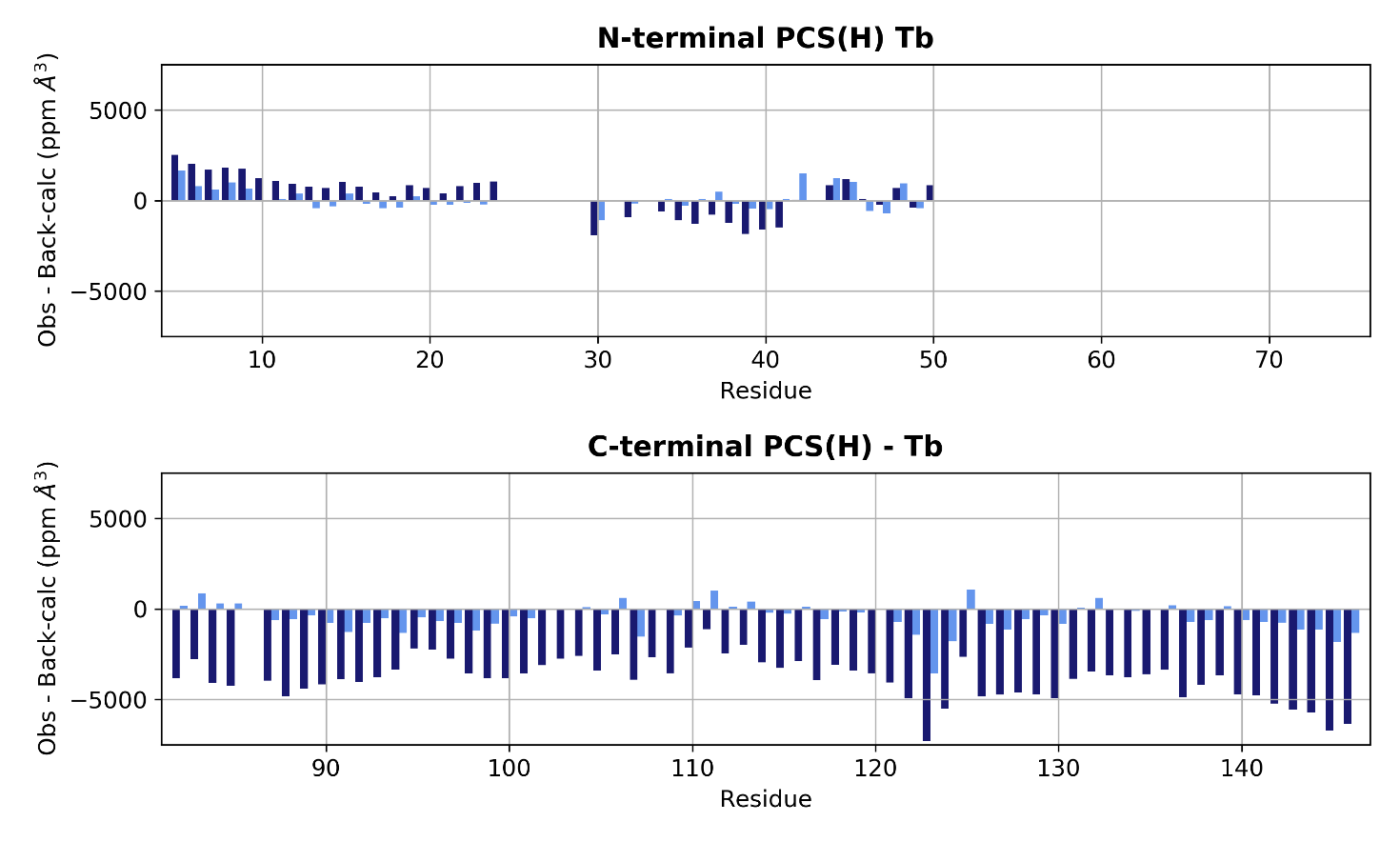


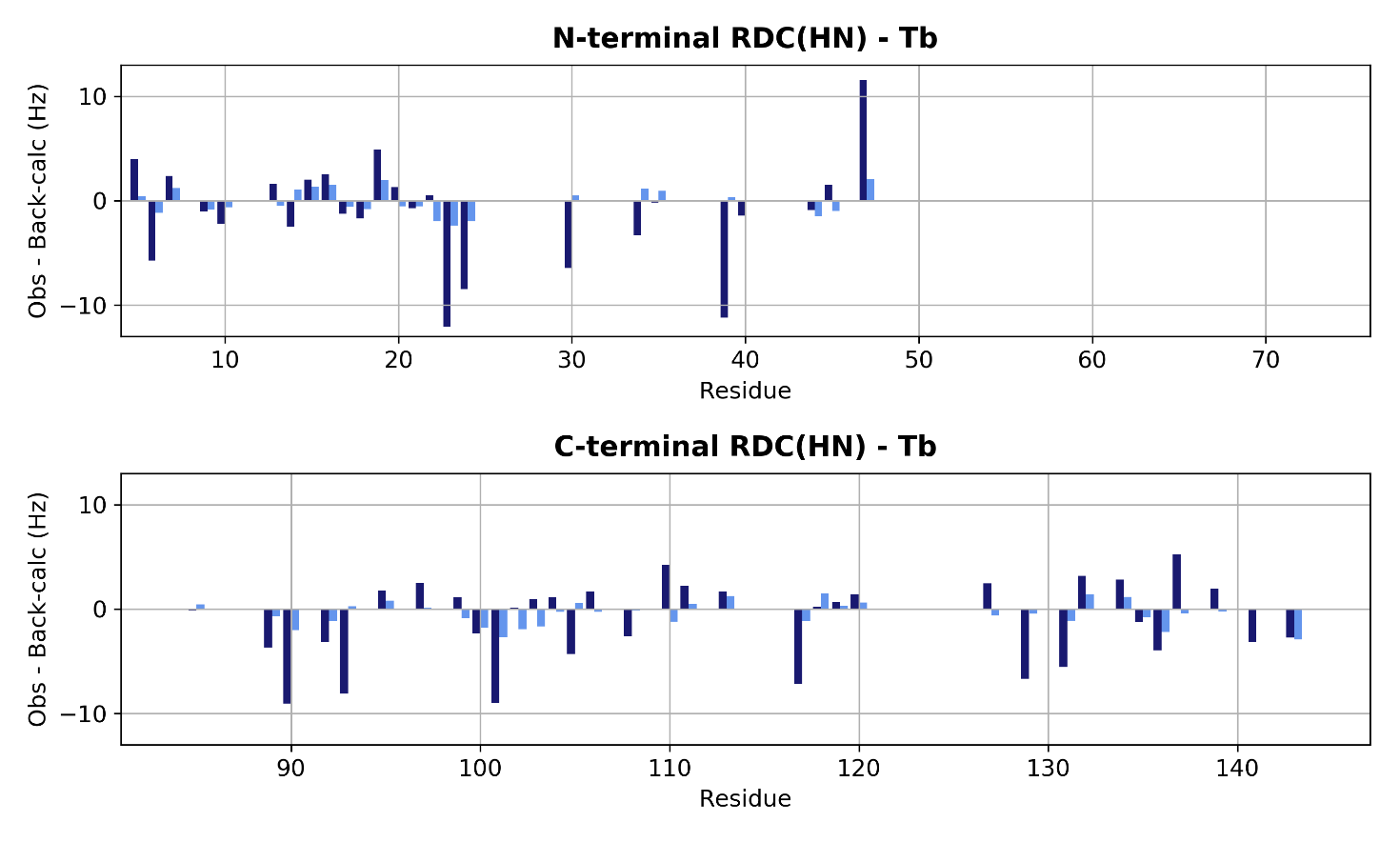


**Figure S3a**: residue-by residue discrepancies calculated for the CaM-IQ peptide (2BE6) for Tb(III) for the structures refined by REFMAC (without NMR) (dark blue) and for the structures refined by REFMAC-NMR using the tensor magnitude constraint after applying rigid body minimization (light blue).

**
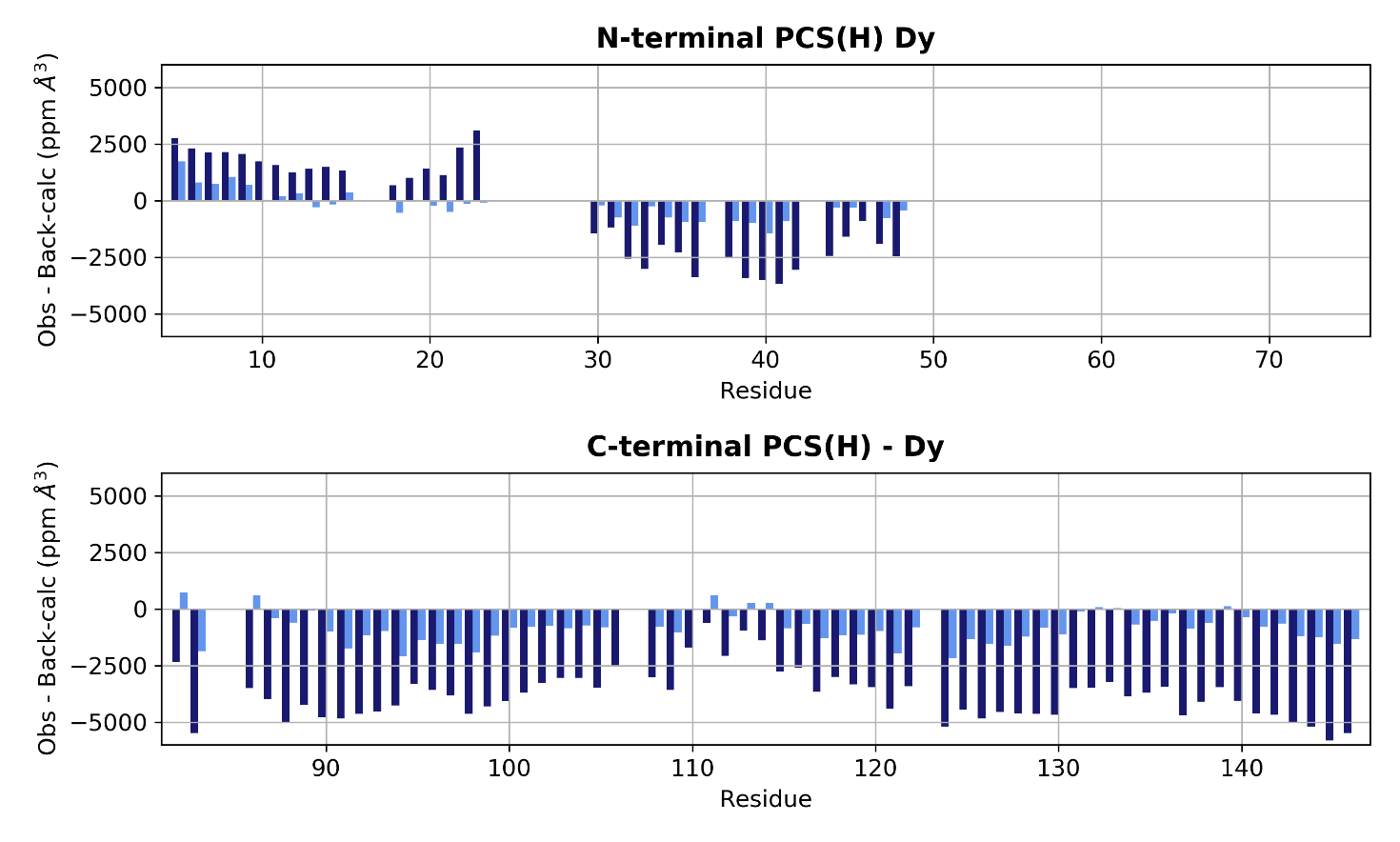
**

**
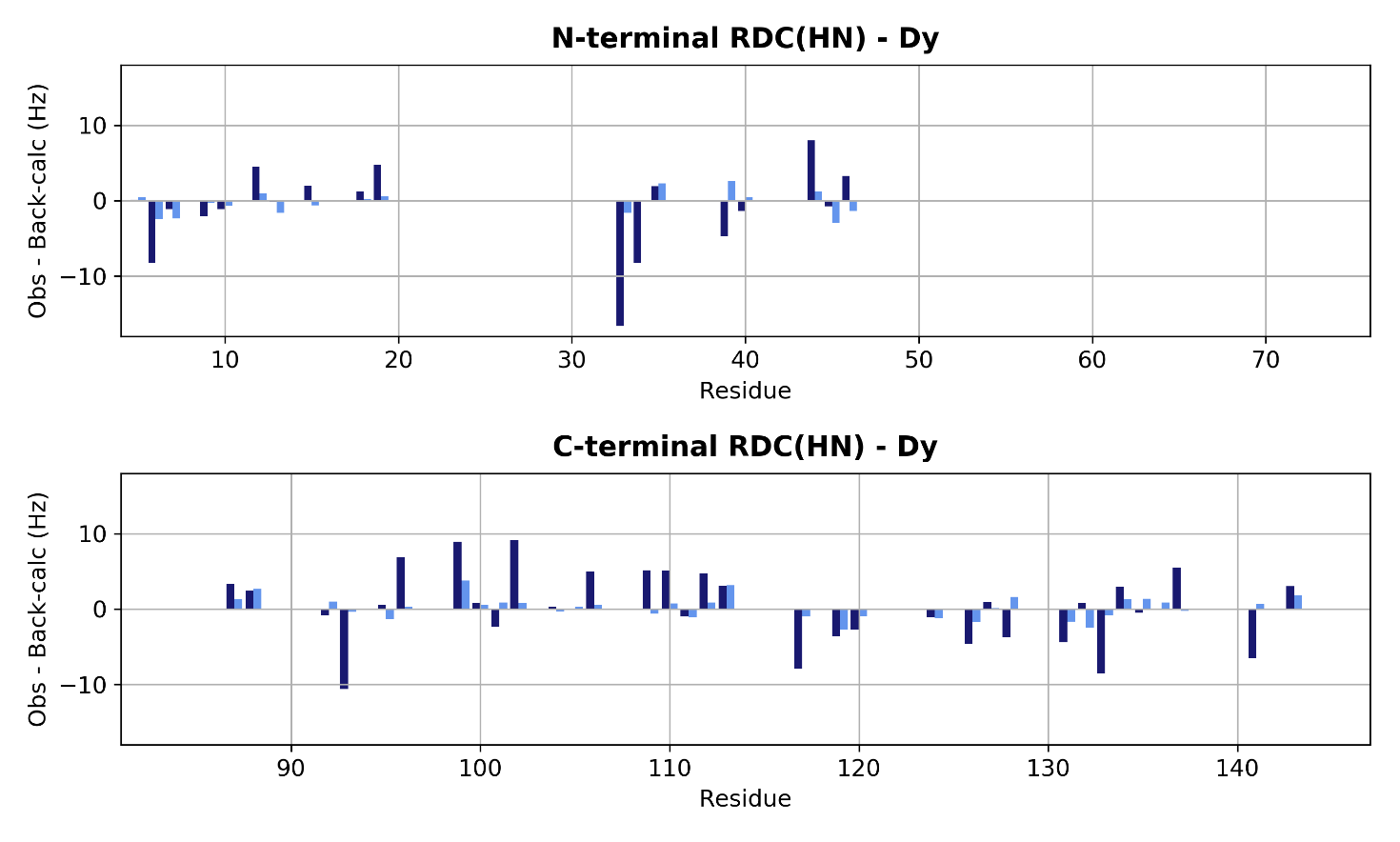
**

**Figure S3b**: residue-by residue discrepancies calculated for the CaM-IQ peptide (2BE6) for Dy(III) for the structures refined by REFMAC (without NMR) (dark blue) and for the structures refined by REFMAC-NMR using the tensor magnitude constraint after applying rigid body minimization (light blue).


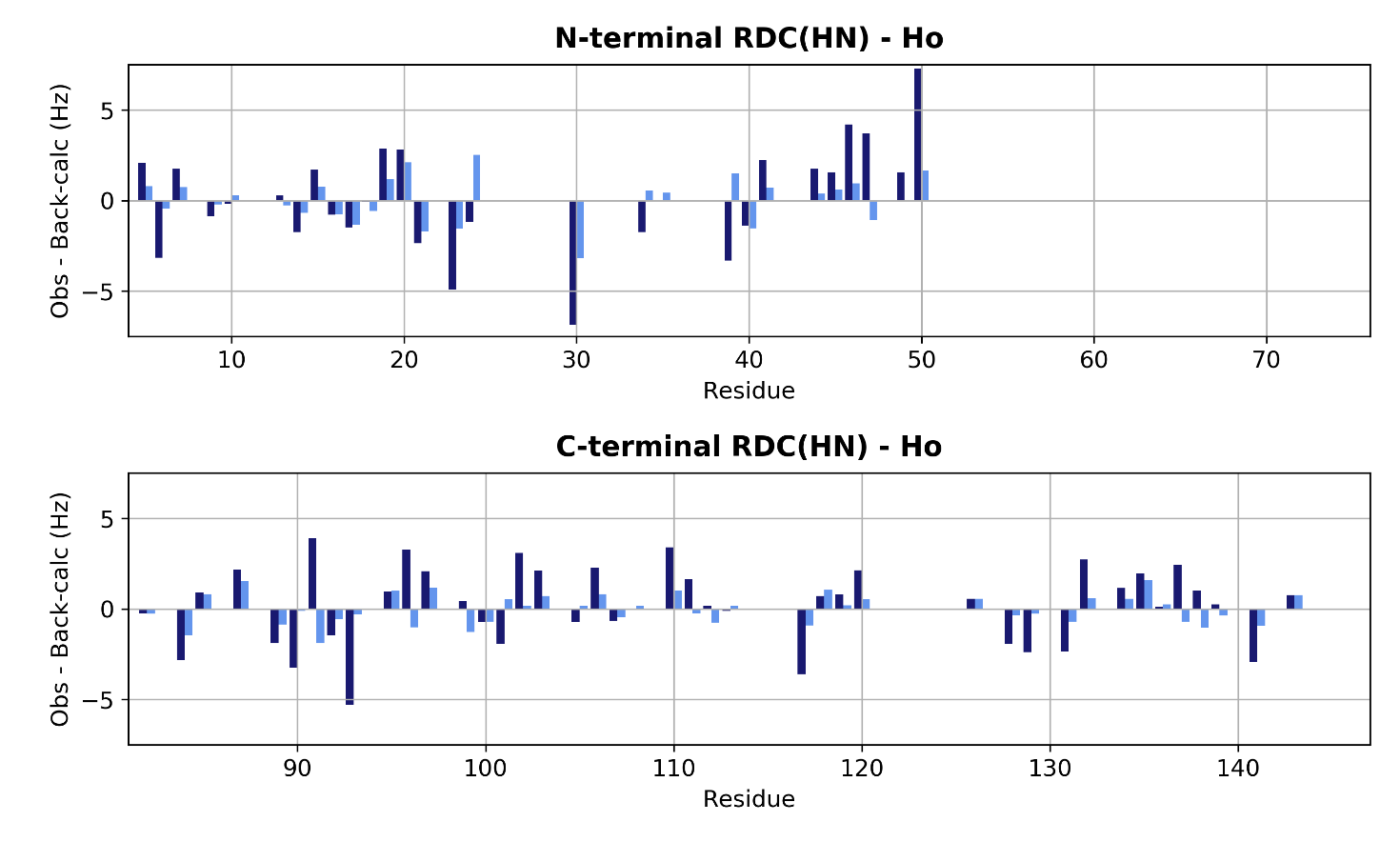

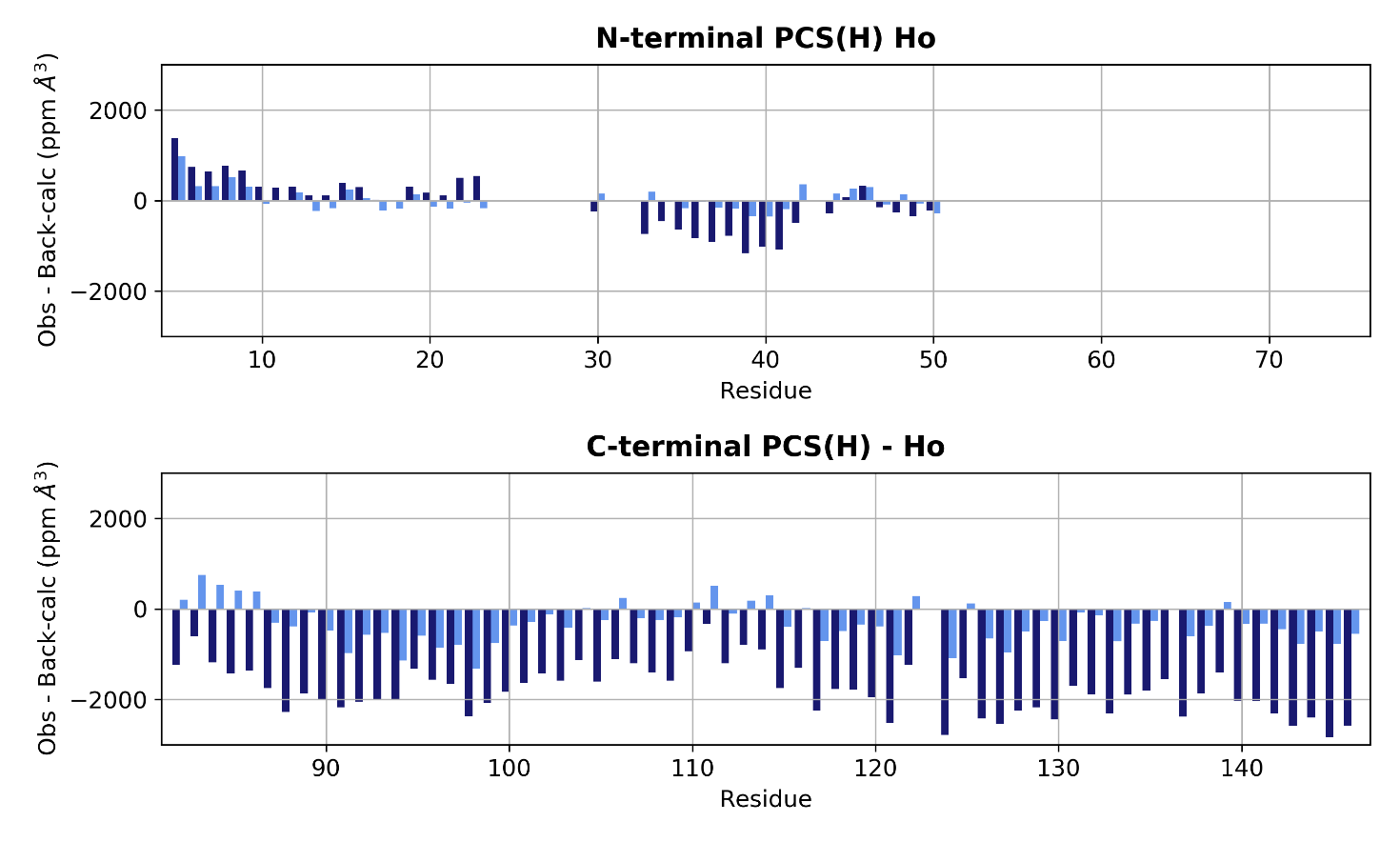


**Figure S3c**: residue-by residue discrepancies calculated for the CaM-IQ peptide (2BE6) for Ho(III) for the structures refined by REFMAC (without NMR) (dark blue) and for the structures refined by REFMAC-NMR using the tensor magnitude constraint after applying rigid body minimization (light blue).

**
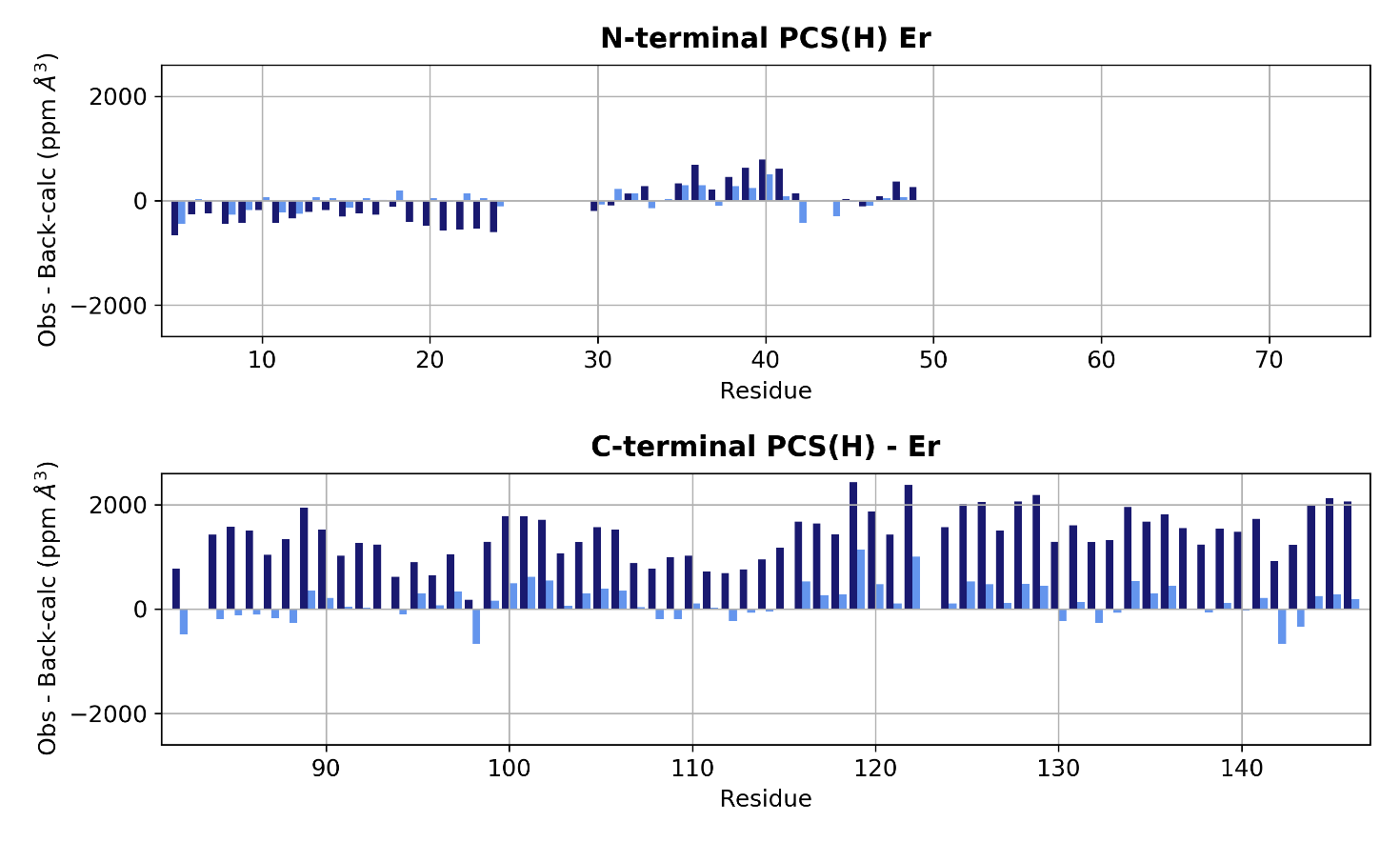
**

**
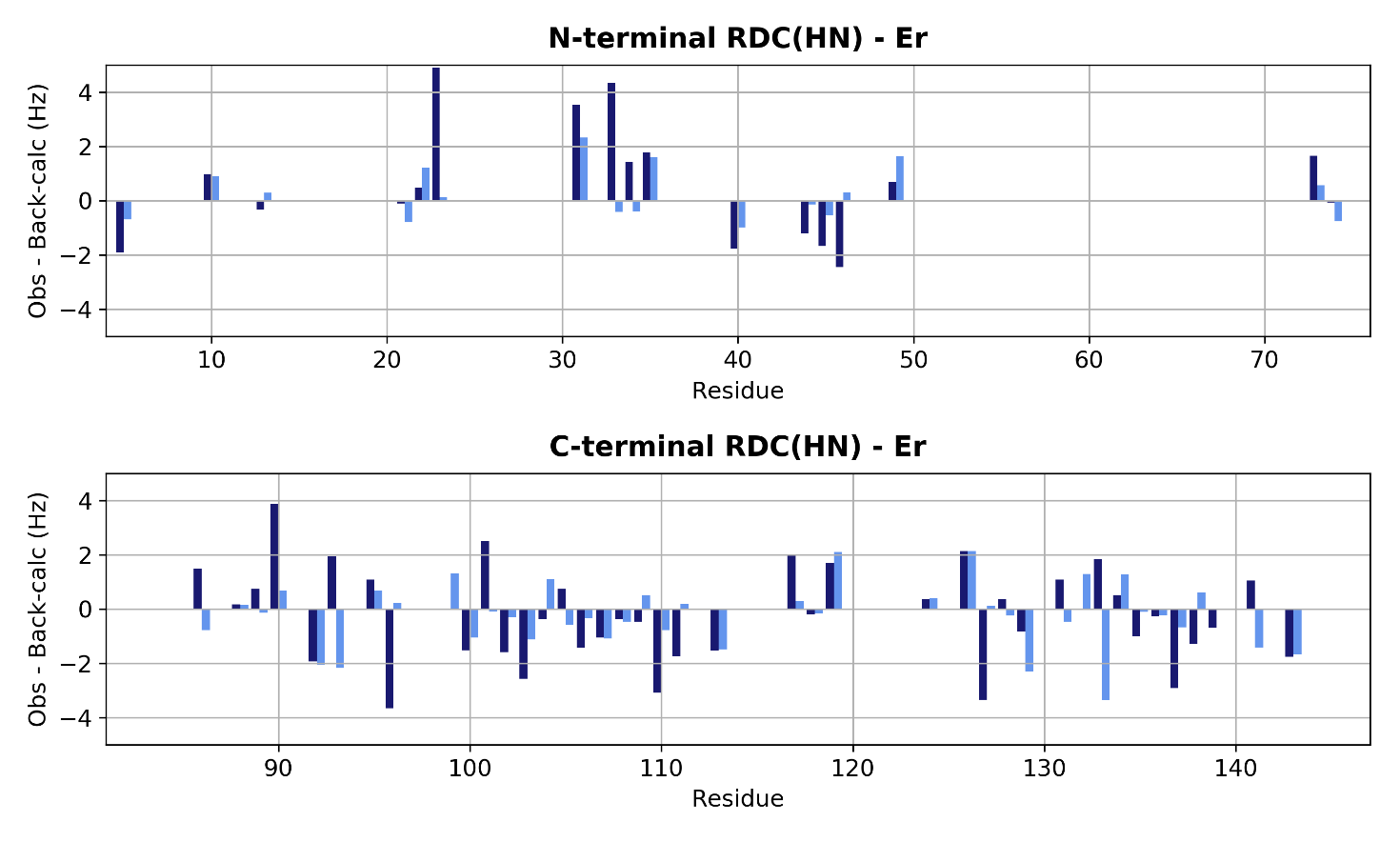
**

**Figure S3d**: residue-by residue discrepancies calculated for the CaM-IQ peptide (2BE6) for Er(III) for the structures refined by REFMAC (without NMR) (dark blue) and for the structures refined by REFMAC-NMR using the tensor magnitude constraint after applying rigid body minimization (light blue).


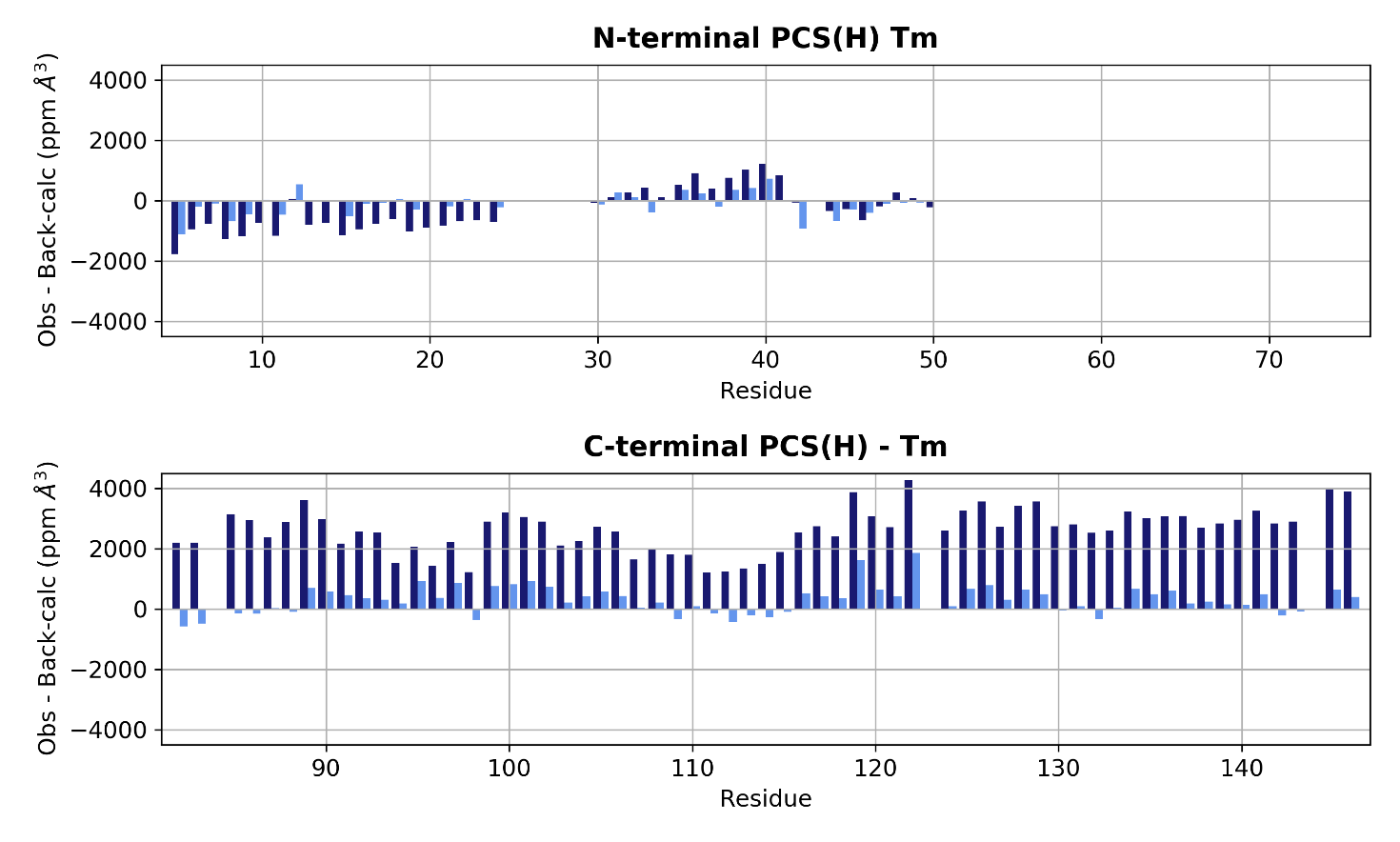


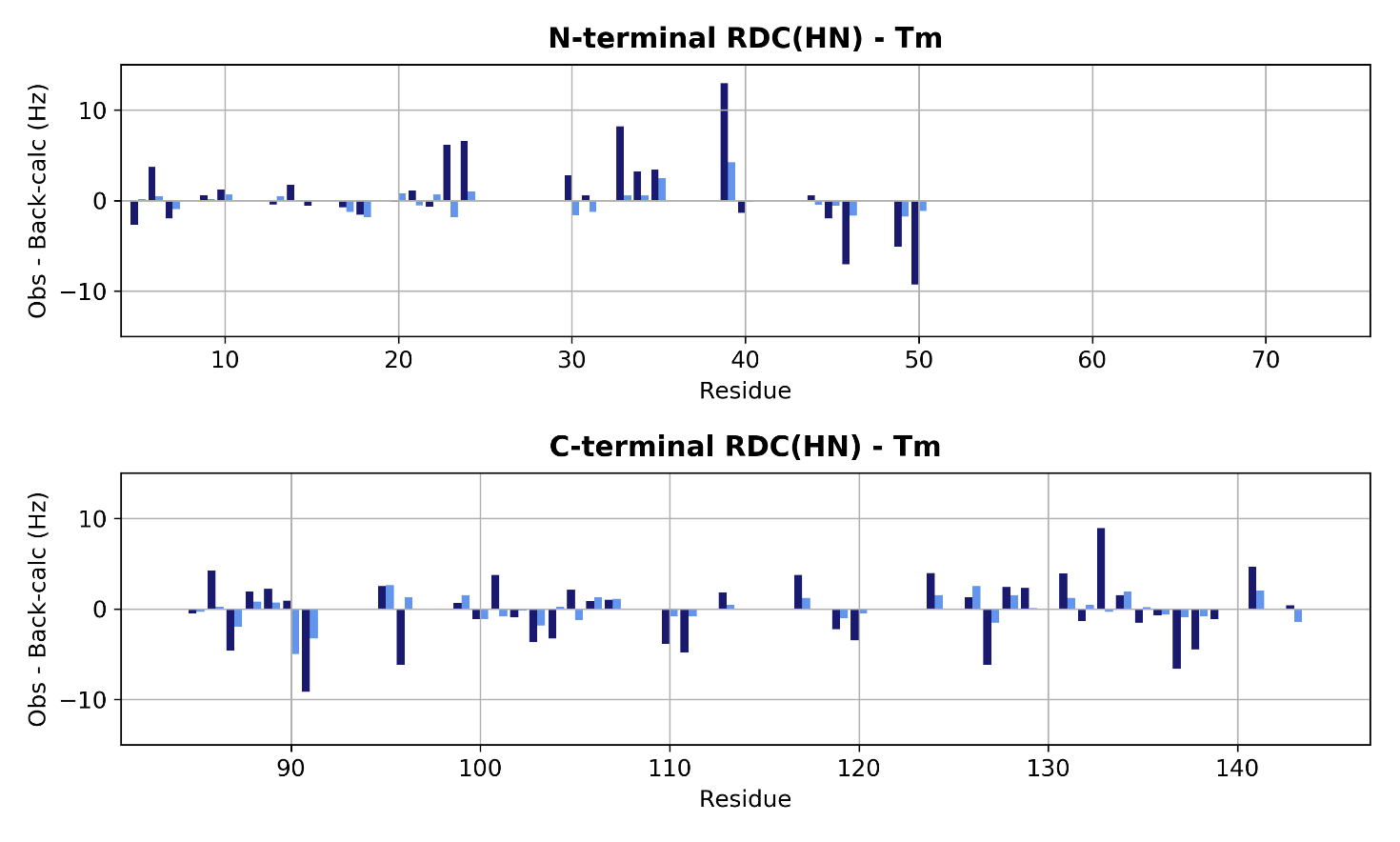


**Figure S3e**: residue-by residue discrepancies calculated for the CaM-IQ peptide (2BE6) for Tm(III) for the structures refined by REFMAC (without NMR) (dark blue) and for the structures refined by REFMAC-NMR using the tensor magnitude constraint after applying rigid body minimization (light blue).


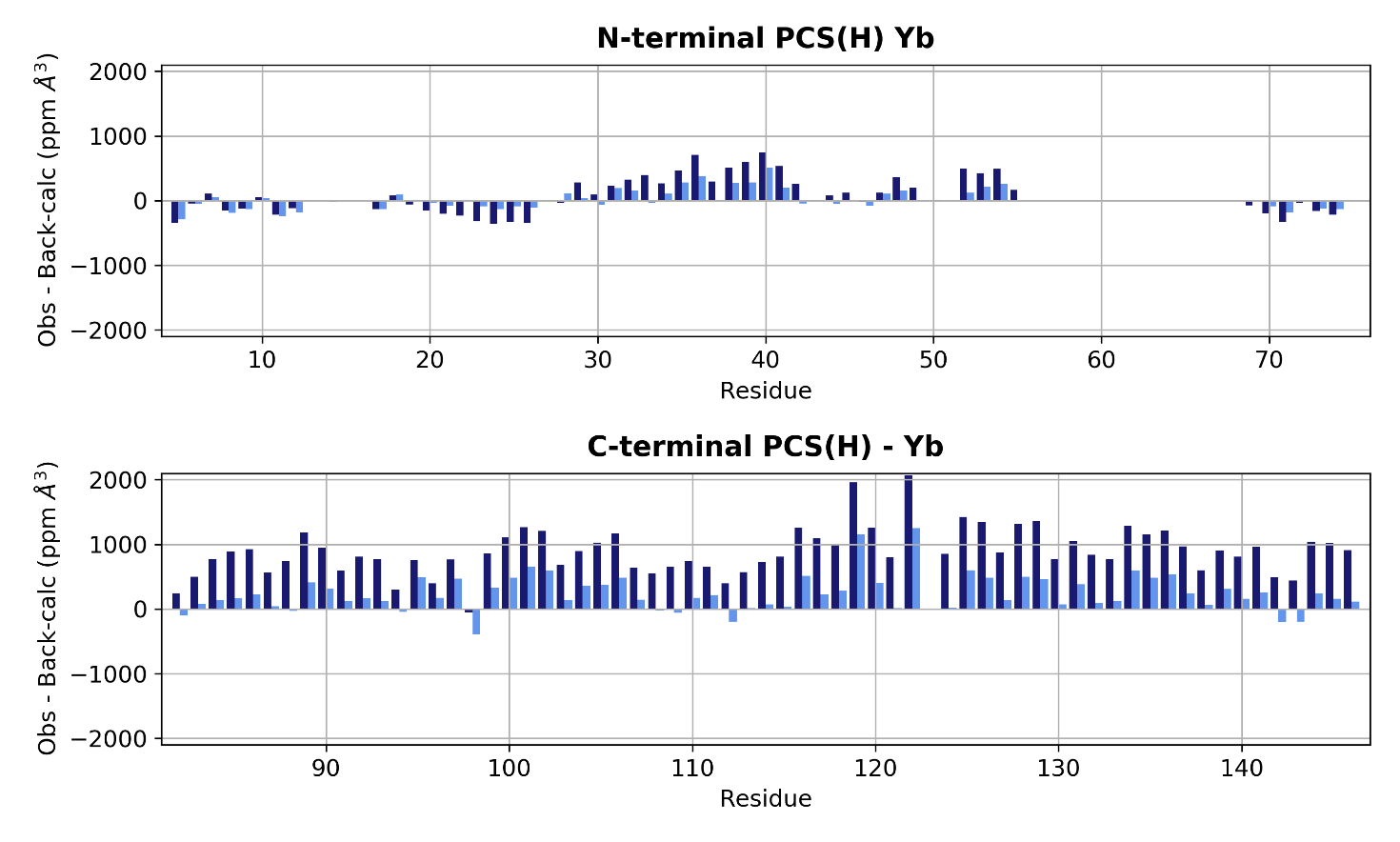


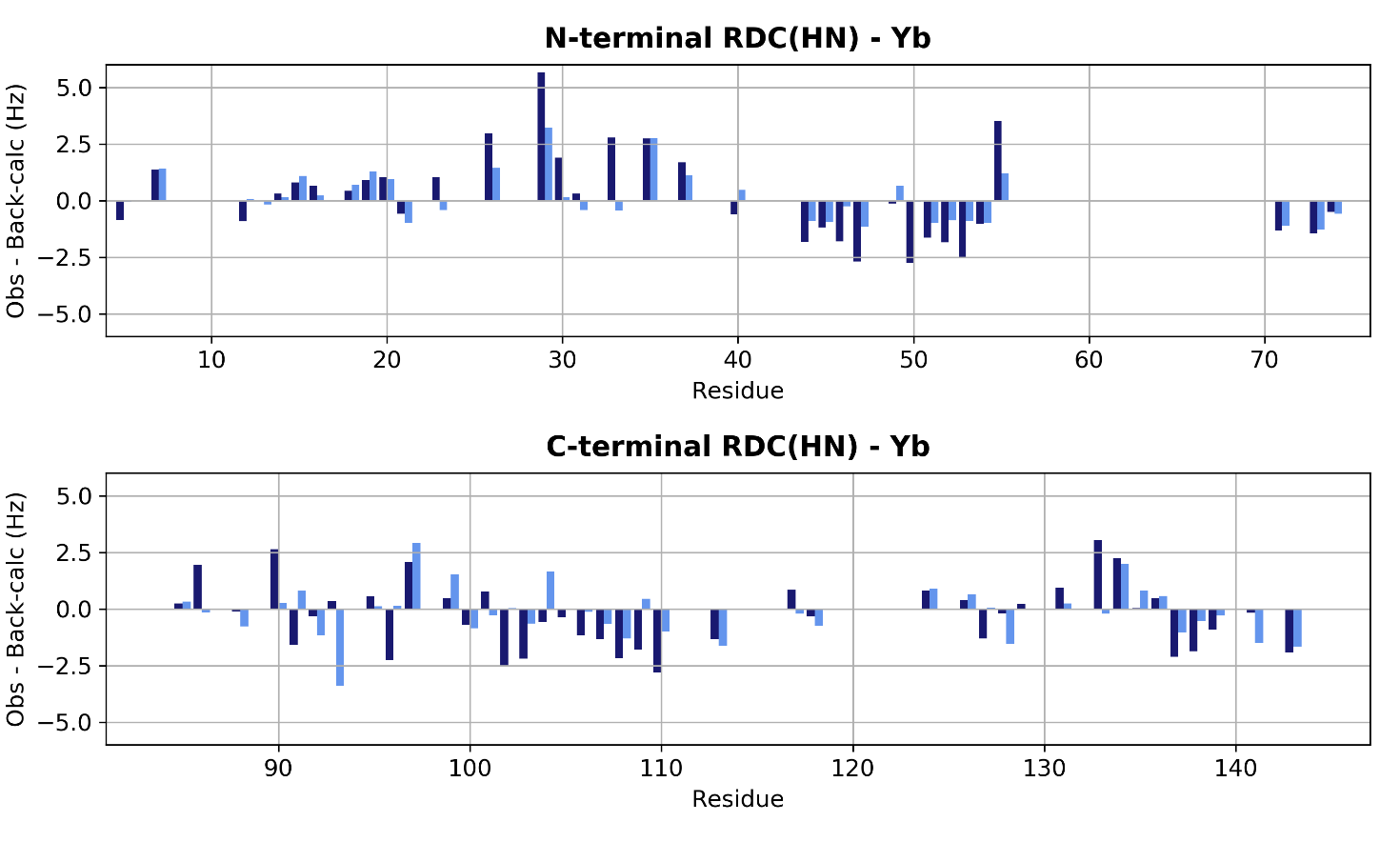


**Figure S3f**: residue-by residue discrepancies calculated for the CaM-IQ peptide (2BE6) for Yb(III) for the structures refined by REFMAC (without NMR) (dark blue) and for the structures refined by REFMAC-NMR using the tensor magnitude constraint after applying rigid body minimization (light blue).


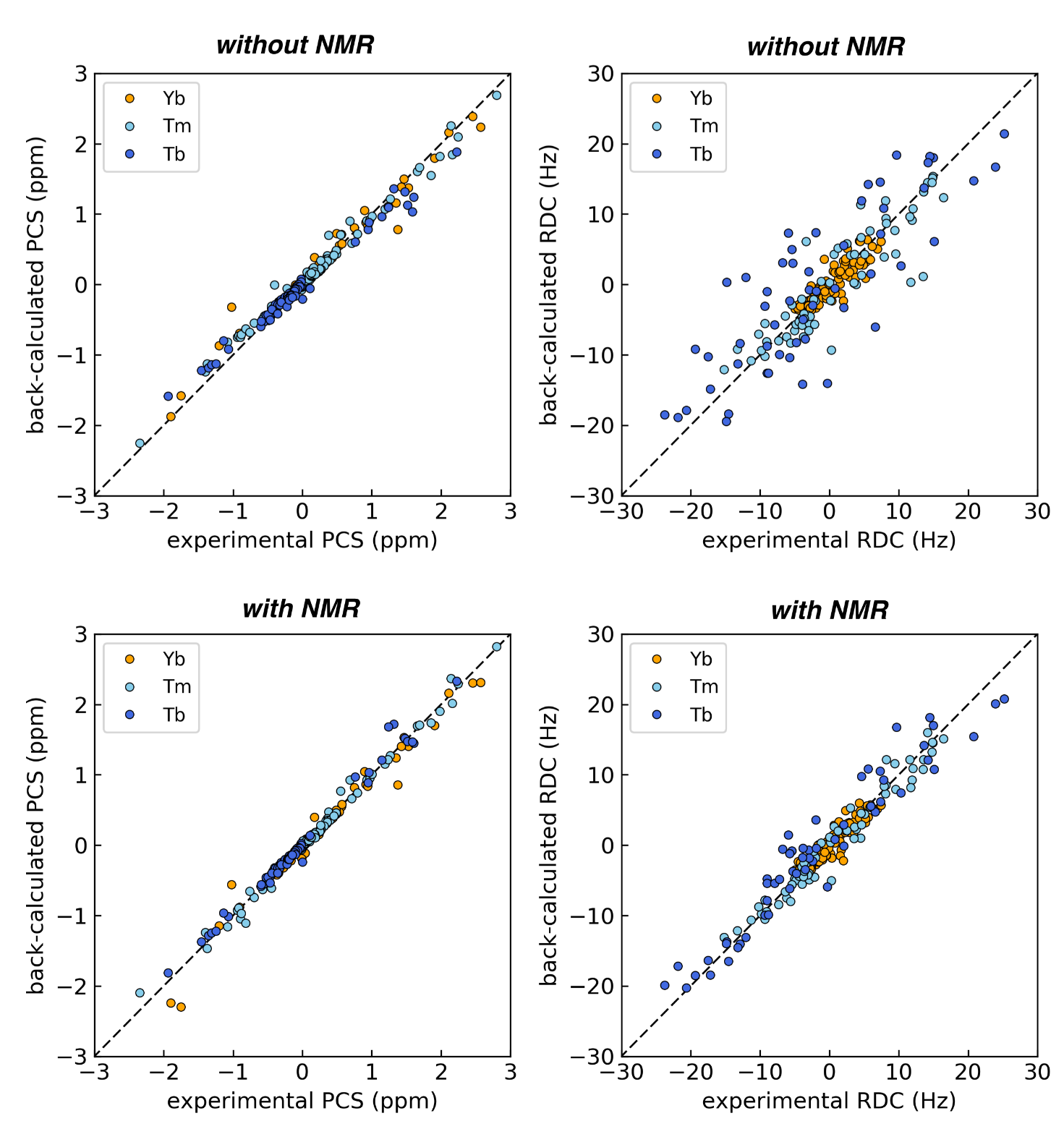


**Figure S4**: Experimental vs back-calculated PCS and RDC data for the CaM-DAPk peptide structure (pdb code 1YR5) refined using REFMAC without and with the inclusion of NMR data.


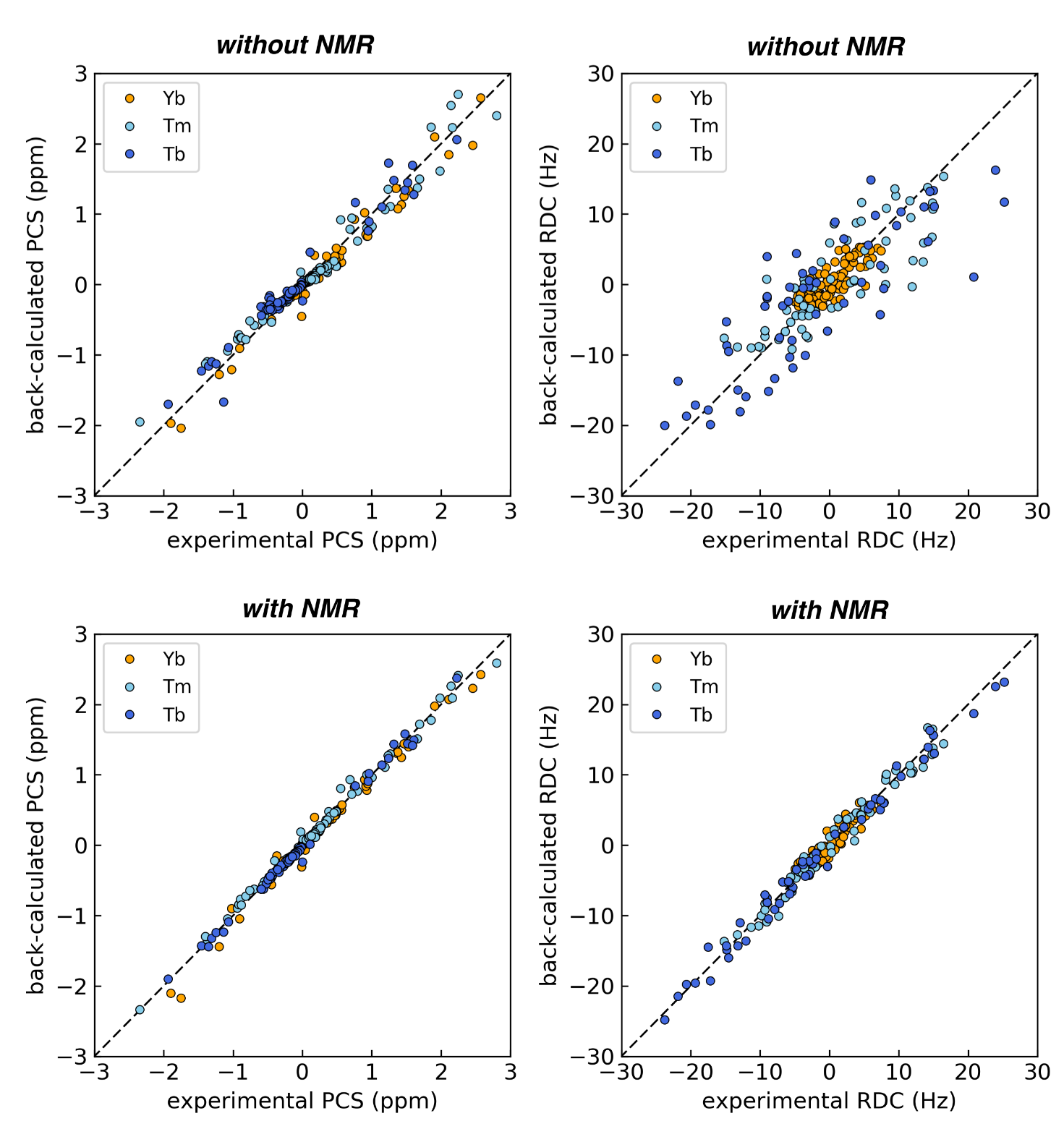


**Figure S5**: Experimental vs back-calculated PCS and RDC data for the CaM-DAPk protein structure (pdb code 2X0G) refined using REFMAC without and with the inclusion of NMR data.


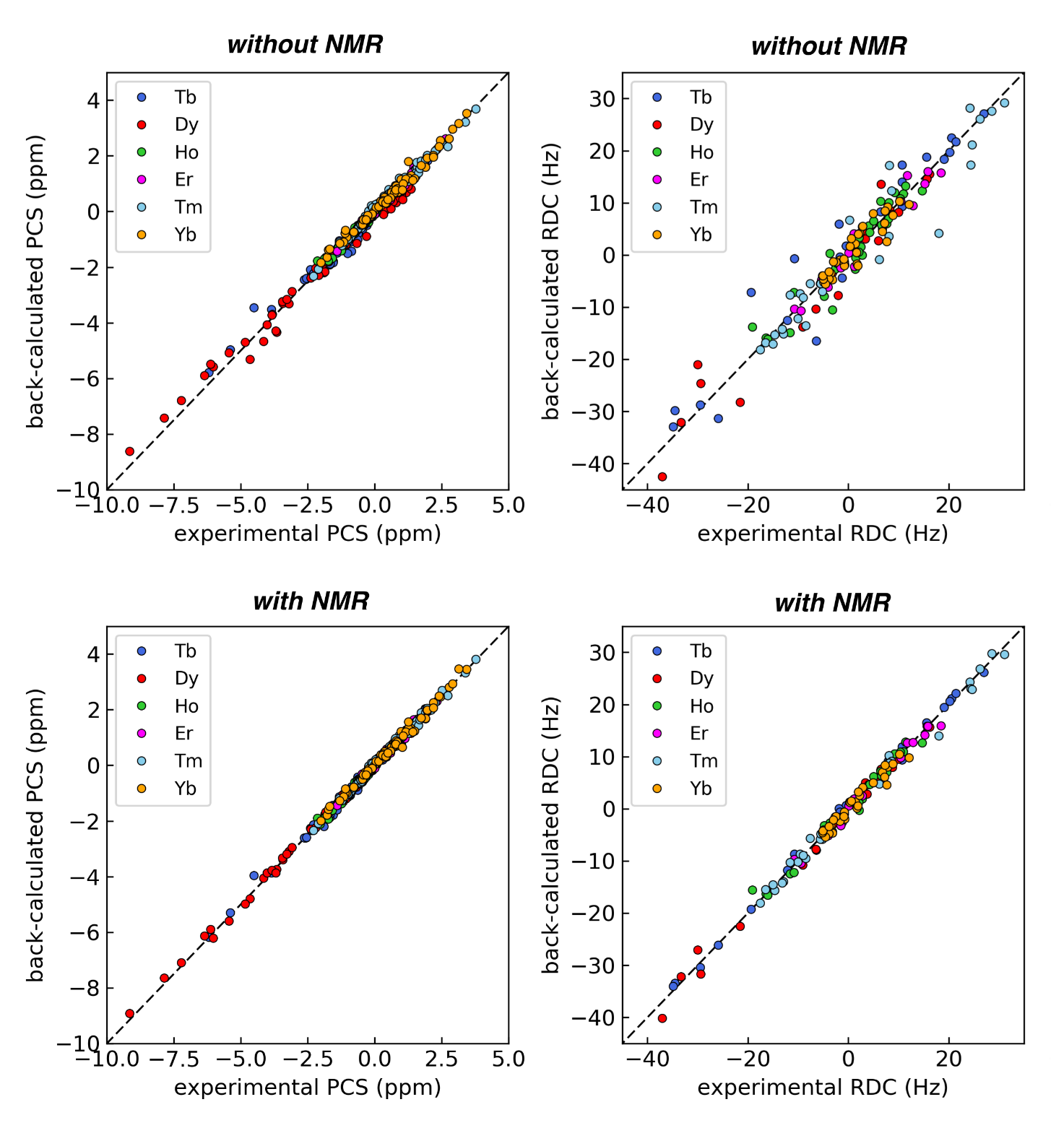


**Figure S6**: Experimental vs back-calculated PCS and RDC data for the CaM-IQ peptide structure (pdb code 2BE6) refined using REFMAC without and with the inclusion of NMR data.

**An instruction file for REFMAC-NMR**

The instructions for the use of REFMAC-NMR are given in the Supporting Information of Rinaldelli M, Ravera E, Calderone V, et al (2014) Acta Crystallogr D 70:958–967. The instruction file for refinement with constraints on tensor properties largely matches the instruction file that would be used for the standard REFMAC-NMR refinement, but an independent set of metals must be created referring to each structural unit. In the case of CaM-DAPk, the three metals (Tb(III), Tm(III), Yb(III)) are thus repeated twice, one for the N-terminal domain and one for the C-terminal domain. Since both PCSs and RDCs were measured, the same procedure is repeated for each type of data, as follows:

nmr metal 1 in PCS res 701 atom TB chain B

nmr metal 2 in PCS res 702 atom TM chain B

nmr metal 3 in PCS res 703 atom YM chain B

nmr metal 4 in PCS res 704 atom TB chain B

nmr metal 5 in PCS res 705 atom TM chain B

nmr metal 6 in PCS res 706 atom YM chain B

nmr metal 1 in RDC res 701 atom TB chain B

nmr metal 2 in RDC res 702 atom TM chain B

nmr metal 3 in RDC res 703 atom YB chain B

nmr metal 4 in RDC res 704 atom TB chain B

nmr metal 5 in RDC res 705 atom TM chain B

nmr metal 6 in RDC res 706 atom YM chain B

Also in this case, all the metals should be defined as “visible” only by NMR (and so invisible by the X-ray diffraction data), as following:

nmr visible only res 701

nmr visible only res 702

nmr visible only res 703

nmr visible only res 704

nmr visible only res 705

nmr visible only res 706

If both experimental PCS and RDC data refer to the same tensor, the “joint” estimation of the tensor is allowed in the following way:

# choose estimation between SEPAR JOINT SCALE:

nmr estimation JOINT

An overall weight and tolerance to the PCS and RDC dataset can also be given. These weights will be multiplied to those already present in the file of experimental data:

nmr rtolerance 1.0

nmr weight for PCS 0.5

nmr weight for RDC 0.5

The only difference is the inclusion of the constraints on magnitude (“magn”), orientation (“orie”) or on both (“both”) of the tensors belonging to the two domains, which are defined as:

nmr constrain magn tensor 1 JOINT chain B tensor 4 JOINT chain B

nmr constrain magn tensor 2 JOINT chain B tensor 5 JOINT chain B

nmr constrain magn tensor 3 JOINT chain B tensor 6 JOINT chain B
